# Reference-free deconvolution of DNA methylation data and mediation by cell composition effects

**DOI:** 10.1186/s12859-016-1140-4

**Published:** 2016-06-29

**Authors:** E. Andres Houseman, Molly L. Kile, David C. Christiani, Tan A. Ince, Karl T. Kelsey, Carmen J. Marsit

**Affiliations:** School of Biological and Population Health Sciences, College of Public Health and Human Sciences, Oregon State University, Corvallis, OR USA; Department of Environmental Health, Harvard T. H. Chan School of Public Health, Boston, MA USA; Department of Pathology, University of Miami, Miller School of Medicine, Miami, FL USA; Department of Epidemiology, Department of Pathology and Laboratory Medicine, Brown University, Providence, USA; Department of Community and Family Medicine, Dartmouth Medical School, Hanover, NH USA

**Keywords:** Deconvolution, DNA methylation, Epigenetics, Non-negative matrix factorization

## Abstract

**Background:**

Recent interest in reference-free deconvolution of DNA methylation data has led to several supervised methods, but these methods do not easily permit the interpretation of underlying cell types.

**Results:**

We propose a simple method for reference-free deconvolution that provides both proportions of putative cell types defined by their underlying methylomes, the number of these constituent cell types, as well as a method for evaluating the extent to which the underlying methylomes reflect specific types of cells. We demonstrate these methods in an analysis of 23 Infinium data sets from 13 distinct data collection efforts; these empirical evaluations show that our algorithm can reasonably estimate the number of constituent types, return cell proportion estimates that demonstrate anticipated associations with underlying phenotypic data; and methylomes that reflect the underlying biology of constituent cell types.

**Conclusions:**

Our methodology permits an explicit quantitation of the mediation of phenotypic associations with DNA methylation by cell composition effects. Although more work is needed to investigate functional information related to estimated methylomes, our proposed method provides a novel and useful foundation for conducting DNA methylation studies on heterogeneous tissues lacking reference data.

**Electronic supplementary material:**

The online version of this article (doi:10.1186/s12859-016-1140-4) contains supplementary material, which is available to authorized users.

## Background

In the last decade, there has been an increasing interest in epigenome-wide association studies (EWAS), which aim to investigate associations between DNA methylation and health or exposure phenotypes across the genome. Numerous publications have reported associations between DNA methylation profiled in a single tissue and disease states or exposure phenotypes. Most of these studies have used whole blood [1] or cord blood [2–4], but some have used other media such as buccal swabs [5], adipose tissue [6, 7], and placenta [8–11].

However, most tissues are complex mosaic of cells derived from at least two and sometimes three different germ layers; endoderm, mesoderm and ectoderm that give rise to both epithelial and stromal compartments. Just the epithelial component of an organ can be composed of many cell types; for example we found that breast epithelium is composed of at least 10–12 cell types [12] with potentially distinct DNA methylation profiles [13]. Added to this complexity are the cells in the stromal component with distinct functions, including vascular and lymphoid endothelial cells and pericytes, immune cells such as macrophages, leukocytes and lymphocytes, stromal fibroblasts, myofibroblasts, myoepithelial cells, as well as adipose cells, endocrine cells, nerve cells and other cellular and tissue elements that have different but systematically varying developmental origins. The complexity of the epigenome in normal tissues has been described in a recent analysis of 111 reference human epigenomes of human tissues [14]. Thus, because normal tissue development, individual cellular differentiation and cellular lineage determination are regulated by epigenetic mechanisms, which include chromatin alterations as well as DNA methylation [15–18], many phenotypic associations with DNA methylation may be explained in whole or in part by systematic associations with the distribution of underlying cell types. This has been demonstrated statistically in numerous papers [19–22] and in one notable recently published manuscript which identified and confirmed the specific cell subtype responsible for the highly replicated relationship between tobacco smoking exposure and DNA methylation of the GPR15 locus [23]. This phenomenon has led to an interest in methods for adjusting EWAS studies for cell-type heterogeneity. In *referenced-based* deconvolution methods, the distribution of cell types is obtained by projecting whole-tissue DNA methylation data onto linear spaces spanned by cell-type-specific methylation profiles for a specific set of CpGs that distinguish the cell types, so-called *differentially methylated positions* (DMPs) [19]; these methods require the existence of a reference set consisting of the cell-type specific methylation profiles, such as those that exist for blood [19, 24, 25]. However, no such reference sets exist for solid tissues of interest, such as adipose and placenta, or even tumors, thus motivating *reference-free* methods [13, 26, 27] that seek to adjust DNA methylation associations for cell-type distribution.

Numerous cell-type deconvolution methods are currently available, many of them based on mRNA or protein expression [28]; all of them are essentially either reference-based, i.e. supervised by the pre-selection of loci known to differentiate cell types, or else reference-free, i.e. essentially unsupervised. While reference-based deconvolution methods allow for direct inference of the relationship between phenotypic variation and altered cell composition of characterized cell subtypes, reference-free approaches can provide only limited, if any, information on the types of cells contributing to the phenotypic association. In this article we propose a simple method for reference-free deconvolution that addresses this challenge and that provides both interpretable outputs – proportions of putative cell types defined by their underlying DNA methylation profiles – as well as a means for evaluating the extent to which the underlying profiles reflect specific types of cells.

**Fig. 1:**
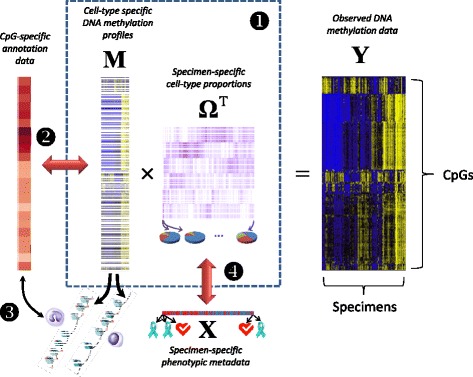
Overview of proposed Methods. If associations between DNA methylation data **Y** and phenotypic metadata **X** factor through the decomposition **Y** = **MΩ** ^*T*^, and the data in **M** serve to distinguish cell types by their associations with relevant annotation data, then associations between **X** and **Y** are explained in whole or in part by differences in the distribution of constituent cell types. Numbers indicate steps in analysis: (1) deconvolution; (2) determining discriminating loci; (3) gene-set analysis; (4) analysis of associations with phenotype

Our fundamental approach is as follows: we assume an *m* × *n* matrix **Y** representing DNA methylation data collected for *n* subjects or specimens, each measured on an array of *m* CpG loci, and that the measured values are constrained to the unit interval [0, 1], each roughly representing the fraction of methylated cytosine molecules in the given sample at a specific genomic position. This conforms to the typical *average beta* output of popular platforms such as the Infinium arrays by Illumina, Inc. (San Diego, CA), i.e. the older HumanMethylation27 (27K) platform, which interrogates 27,578 CpG loci, and the newer HumanMethylation450 (450K) platform, which interrogates 485,412 CpG loci; however, it also conforms to the results of sequencing-based platforms such as whole genome bisulfite sequencing (WGBS). In reference-based methods, the following relation is assumed to hold: **Y** = **MΩ**^*T*^, where **M** is a *known m* × *K* matrix representing *m* CpG-specific methylation states for *K* cell types and **Ω** is an *n* × *K* matrix representing subject-specific cell-type distributions (each row representing the cell-type proportions for a given subject, i.e. the entries of **Ω** lie within [0, 1] and the rows of **Ω** sum to values less than one). Reference-free methods attempt to circumvent lack of knowledge about **M** either by using a two-stage regression analysis (e.g. the Houseman approach [27]) or else fitting a high-dimensional mixed-effects model and equating the resulting random coefficients with cell-mixture effects (i.e. the Zou approach [26]); both methods rely on a predetermined model positing associations between DNA methylation **Y** and phenotypes **X**. For example, the Houseman method posits the model **Y** = **AX**^*T*^ + **R**, where **X** is an *n* × *d* design matrix of phenotype variables and potential confounders; the *m* × *d* regression coefficient matrix **A** and the *m* × *n* error matrix **R** are both assumed to have further linear structure involving **M**, and the common variation between **A** and **R** is assumed to represent systematic association with cell type distribution. However, results of this approach are somewhat influenced by the choice of the dimension of the linear subspace of [**A**, **R**] representing the common variance induced by **M** [20]; consequently there has been recent concern that the method may over-adjust for cell distribution. A similar problem exists with the Zou approach, which models the phenotype as a linear function of DNA methylation, and in which the choice of a tuning parameter can influence the extent to which phenotypic associations are putatively explained by heterogeneity in underlying cell types. Here, we propose that a variant of non-negative matrix factorization be used to decompose **Y** as **Y** = **MΩ**^*T*^, where the entries of the unknown matrices **M** and **Ω** are constrained to lie in the unit interval and the rows of **Ω** are constrained to sum to a value less than or equal to one. This approach is similar to existing approaches for estimating the proportion of normal tissue cells in a tumor sample or otherwise deconvolving mixtures of cells [29–33]. Additionally, this factorization conforms to the biological assumption that DNA methylation measurements **Y**, regardless of associated metadata **X**, ultimately arise as linear combinations of constituent methylomes, as we have previously argued [20]. However, such constrained factorizations can be computationally intensive, and it is still necessary to specify the number *K* of assumed cell types, so in Additional file 1: Section S1 we propose a fast approximation that facilitates resampling, which is the basis of our method for determining *K*, described in Additional file 1: Section S2. Note that *K* = 1 corresponds to the case where there are no relevant constituent cell types, which should be true for relatively pure media. If associations remain between **Ω** and **X**, i.e. if the associations between **X** and **Y** factor through the decomposition **Y** = **MΩ**^*T*^, then these associations are potentially explained by systematic changes in cell composition. Evidence for mediation of associations by cell type is substantially strengthened if the methylomes represented by **M** map to biological processes that correspond to distinct populations of cells. To that end, we propose a simple companion analytical procedure for the interpretation of the methylomes represented by **M**. Denote each row of **M** (corresponding to one CpG) as the *K* × 1 vector **μ**_*j*_, *j* ∈ {1, …, *m*}. CpG loci that most differentiate the *K* putative cell types will tend to have distinct values within **μ**_*j*_; thus high values of the row-variance *s*_*j*_^2^ = var{*μ*_*j*1_, …, *μ*_*jK*_} should correspond to CpGs that are most relevant to the biological distinctions among the *K* cell types, and this can be tested with auxiliary annotation data. Figure 1 illustrates our approach.

We demonstrate these methods in analyses of 23 genome-scale DNA methylation data sets from 13 distinct data collection efforts, including four blood data sets, several breast tumor data sets (including data from The Cancer Genome Atlas, *TCGA*), vascular and liver tissues, sperm, and four separate media collected on the same population, Bangladeshi neonates, including placenta. In addition, we leverage data derived from The Roadmap Epigenomics Project, demonstrating their utility in addressing the biological relevance of fitted methylomes **M**.

## Results

To test our proposed approach, we analyzed 23 DNA methylation datasets from 13 distinct studies, each set of DNA methylation measurements obtained via the Infinium 27K or 450K platform. Four blood data sets [3, 22, 34, 35] were included as positive controls (given the existing reference data and known heterogeneity), each collected in the context of an epidemiologic study, and each assumed to exhibit heterogeneity in cell type as previously described [3, 19, 22]. Sperm [36] and isolated vascular tissues [37] were included as negative controls, presumed to represent relative homogeneity in terms of constituent cell types. Note that four datasets arose from one study on arsenic exposure in Bangladeshi neonates [3, 9], in which four separate tissues were obtained from the same individuals. Also included were arterial tissue [38], liver tissue [39], and data from cancer data sets [40–43], including breast tissues from *TCGA* [44]. Table 1 lists the data sets, their sources and their short descriptions. Additional file 1: Figure S3.1 shows the results of hierarchical clustering applied to 26,476 CpG sites common across the datasets (Manhattan distances based on mean methylation for the data set, clustering based on Ward’s method implemented as *Ward.D* in R version 3.2.2). Additional file 1: Figure S3.2 summarizes the number of CpGs analyzed for each data set, by fraction of samples observed for each CpG. Note the strong clustering of data sets by type of media. The ordering of data sets in Table 1 and many subsequent figures are based on the clusters shown in Additional file 1: Figure S3.1.

**Table 1:** Summary of Datasets

Code	Tissue	Source	Ref	Platform	Source description	Number	Covariate model
g[nt]	gastric tissue: tumor + normal	GEO: GSE30601	[42]	27K	203 gastric tumors and 94 matched gastric non-malignant samples.	297	Tumor[normal|tumor]
g[n]	gastric tissue: normal					94	–
g[t]	gastric tissue: tumor					203	–
br-1[t]	breast: tumor	GEO: GSE20712	[43]	27K	119 breast tumor samples with histological information. Removed 29 samples with ambiguous or missing histology.	119	Histology[basal|HER2|LumA|LumB] + Age[young|old] + Size[small|large]
br-2[t]	breast: tumor	GEO: GSE31979	[40]	27K	103 primary invasive breast tumors.	90	Histology[basal|ER-|ER+|HER2|LumA|LumB] + Age
br-3[t]	breast: tumor	GEO: GSE32393	[41]	27K	Breast tumor samples: 91 invasive ductal, 13 invasive lobular, 10 mucinous or medullary; 76 were ER+.	114	ER[ER-|ER+] + Histology[duct|lob|muc or med] + Age
bl-ov	peripheral blood	GEO: GSE19711	[35]	27K	Whole blood from 131 ovarian cancer cases (drawn pre-treatment) and 274 controls.	402	Case[control|ovarian cancer case] + Age
bl-hn	peripheral blood	GEO: GSE30229*	[34]	27K	Peripheral blood from 92 head and neck squamous cell carcinoma (HNSCC) patients and 92 controls. Removed 2 outlier cases.	182	Case[control|HNSCC case] + Age
BL-ra	peripheral blood	GEO: GSE42861	[22]	450K	Peripheral blood from 354 rheumatoid arthritis patients and 335 controls.	689	Case[control|arthritis case]
BL-as	cord blood	(not public)	[3]	450K*	Cord blood from 45 Bangladeshi neonates, with corresponding drinking water arsenic concentrations.	45	Log-arsenic + Sex[female|male]
SP	sperm	GEO: GSE47627	[36]	450K	26 normal sperm samples.	26	Fraction[swim down|swim up|whole 1h|whole 2h]
BV+LV	endothelial tissue					16	Source[BV|LV]
BV	endothelial tissue: blood vessel	GEO: GSE34487	[37]	450K	16 vascular samples: 6 primary blood vessel endothelial cell samples and 10 primary lymphatic endothelial cell samples.	6	–
LV	endothelial tissue: lymphatic vessel					10	–
UV-as	umbilical vein endothelial tissue	(not public)	[9]	450K*	Umbilical vein endothelial tissues from 51 Bangladeshi neonates, with corresponding drinking water arsenic concentrations.	51	Log-arsenic + Sex[female|male]
AR-as	placental artery	(not public)	[9]	450K*	Placental arteries from 46 Bangladeshi neonates, with corresponding drinking water arsenic concentrations.	46	Log-arsenic + Sex[female|male]
AR[np]	arterial tissue: atherosclerotic + normal	GEO: GSE46394	[38]	450K	15 normal aortic tissues, 15 atherosclerotic aortic lesions, 19 carotid atherosclerotic samples.	49	Source[normal|ath|carotid ath] + Sex[female|male] + Age
AR[n]	arterial tissue: normal aorta					15	^−^
PL-as	placenta	(not public)	[9]	450K*	Placentas from 45 Bangladeshi neonates, with corresponding drinking water arsenic concentrations.	45	Log-arsenic + Sex[female|male]
L[np]	liver tissue: cirrhotic + normal	GEO: GSE60753	[39]	450K	34 normal liver tissues, 21 cirrhotic tissues (due to alcoholism), 45 cirrhotic tissues [due to chronic hepatitis B (HBV) or C (HCV) viral	100	Source[normal|CirrEtOH|CirrV]
L[n]	liver tissue: normal					34	–
BR-tcga[n]	breast: normal	TCGA (11/2014)	[44]	450K*	96 normal breast tissues (matched to tumor) from The Cancer Genome Atlas, downloaded Nov. 2014	96	Age + Race[white|other]
BR-tcga[t]	breast: tumor			450K*	725 breast tumors from The Cancer Genome Atlas, downloaded Nov. 2014	725	Age + Race[white|other] + Staging[II+|III+|IV/X|?] + ER[ER+|ER-] + HER2[HER2+|HER2-|HER2?]

*Processed from idat files using FunNorm algorithm (Bioconductor library minfi). See Methods for details

### Estimated numbers of cell types

**Fig. 2:**
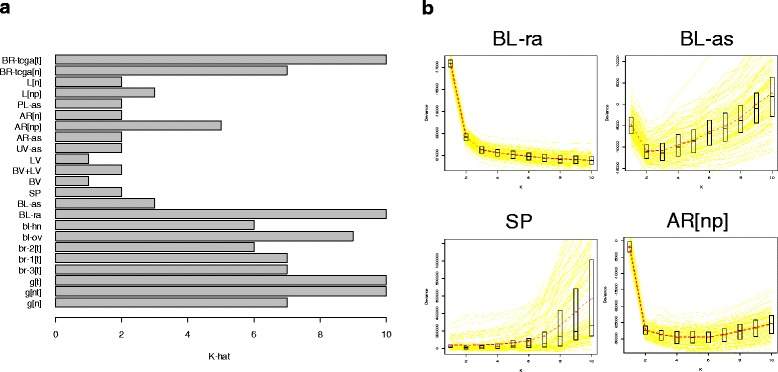
Selection of Number of Classes K. **a** Estimated number $\widehat{K}$ of classes for each data set. **b** Bootstrapped deviance profiles for four selected data sets, along with mean deviance, median deviance, and quartiles for each value of *K*

Using the method described in Additional file 1: Section S2 with 25 iterations, for each data set we found the decomposition **Y** = **MΩ**^*T*^, for values of *K* varying from 2 to either *K*_max_ = 10 or the maximum possible given the sample size (*K*_max_ = 8 for *BV+LV*, 2 for *BV* and *LV*, 7 for *AR[n]*). We then used our bootstrap approach (Additional file 1: Section S3) for determining the number $\widehat{K}$ of classes for each data set, displayed in Fig. 2a, which demonstrates heterogeneity in the number of classes $\widehat{K}$ estimated. $\widehat{K}\ge 3$ for blood data sets ($\widehat{K}=3$ for the cord blood data set *BL-as*, larger for the other three peripheral blood datasets). For breast tissues (both tumor and normal) $\widehat{K}$ was typically large. $\widehat{K}\ge 3$ for the artery and liver data sets having three distinct sources each (*AR[np]* and *L[np]*). $\widehat{K}=1$ for the pure blood and lymphatic vessel data sets (*BV* and *LV*); $\widehat{K}=2$ for other vessel data sets consisting of normal tissue (*AR[n]*, *AR-as*, *BV+LV*, *UV-as*), for the normal liver data set *L[n]*, for sperm (*SP*) and for placenta (*PL-as*). We remark that $\widehat{K}$ was typically lower for datasets that were more likely to be comprised of homogeneous tissues. We also remark that our proposed method of selecting $\widehat{K}$ is based on minimizing a bootstrapped deviance statistic, and that the variation of this statistic across values of *K* can be informative. For example, with the *BL-ra* dataset, the deviance dropped precipitously from *K* = 1 to *K* = 3, while for the sperm data set the deviance remained flat from *K* = 1 to *K* = 6 before rapidly increasing (Fig. 2b).

### Associations with phenotypic metadata

**Fig. 3:**
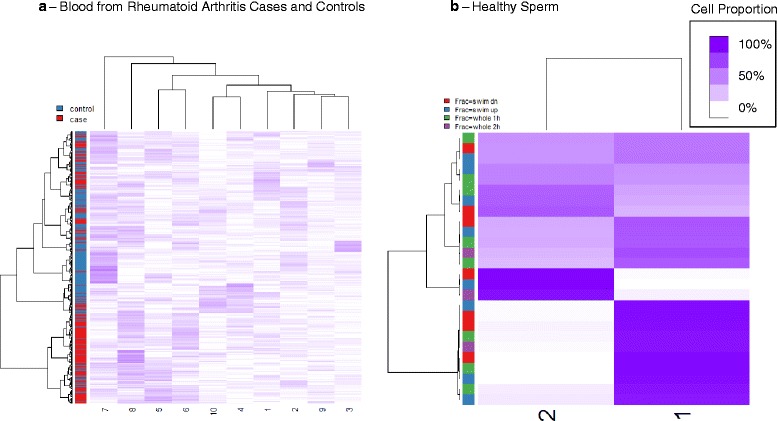
Cell Proportion Matrices. Clustering heatmaps of cell proportion matrix **Ω** for two data sets; purple intensity indicates cell proportion. **a** Blood from rheumatoid arthritis cases and controls (*BL-ra,* $K=\widehat{K}=10$); clustering heatmap obtained from untransformed coefficients and using Ward’s method of clustering (“ward.D” in R *hclust* function). **b** Sperm (*SP*, $K=\widehat{K}=2$)

To examine the associations between **Ω** and various metadata associated with the subjects/specimens in the corresponding study, we fit a quasi-binomial model for each row of **Ω**; Table 1 provides the covariate model **X** used for each data set. As described below and in detail in Additional file 1: Section S4, to circumvent dependence of results on the choice of *K*, we examined associations over the range *K* ∈ {1, …, *K*_max_}, using a permutation test (1000 permutations) for inference on each covariate. Table 2 provides a summary of permutation test results. As shown in Table 2, cell mixture proportions **Ω** were typically significantly associated with major phenotypes of interest and occasionally with age (e.g. *bl-hn* and *BR-tcga[t]*); the exception was the sperm dataset, for which **Ω** was not significantly associated with fraction. Note in particular that for breast tumors, ER status variables (or histology variables incorporating ER status) were significantly associated with **Ω**. Sex was typically not significantly associated with **Ω**. As shown in Fig. 2, the associations between **Ω** and phenotype could be quite striking (e.g. rheumatoid arthritis status) or completely lacking (e.g. sperm fraction). Figure 3a shows clustering heatmap of **Ω** ($K=\widehat{K}=10$) for *BL-ra*, one of the positive control blood data sets, with annotation track showing the associated phenotype rheumatoid arthritis case/control status. Figure 3b shows a similar clustering heatmap for the negative control *SP* (sperm, $K=\widehat{K}=2$), along with the associated phenotype, specimen fraction. Other clustering heatmaps are provided supplementary files.

**Table 2:** Inference with Phenotypic Metadata

Data set	Permutaton *P*-values
g[nt]	Tumor < 0.001
br-1[t]	Histology < 0.001; Age = 0.059; Size = 0.016
br-2[t]	Histology < 0.001; Age = 0.06
br-3[t]	ER < 0.001; Histology = 0.295; Age = 0.008; BSC = 0.297
bl-ov	Case < 0.001; Age = 0.999
bl-hn	Case < 0.001; Age < 0.001
BL-ra	Case < 0.001
BL-as	Log-arsenic < 0.001; Sex = 0.263
SP	Fraction = 0.994
BV+LV	Source = 0.013
UV-as	Log-arsenic = 0.515; Sex = 0.962
AR-as	Log-arsenic = 0.285; Sex = 0.505
AR[np]	Source < 0.001; Sex = 0.043; Age = 0.377
PL-as	Log-arsenic = 0.006; Sex = 0.451
L[np]	Source < 0.001
BR-tcga[n]	Age = 0.089; Race = 0.153
BR-tcga[t]	Age < 0.001; Race < 0.001; Staging = 0.013; ER < 0.001; HER2 < 0.001

**Fig. 4:**
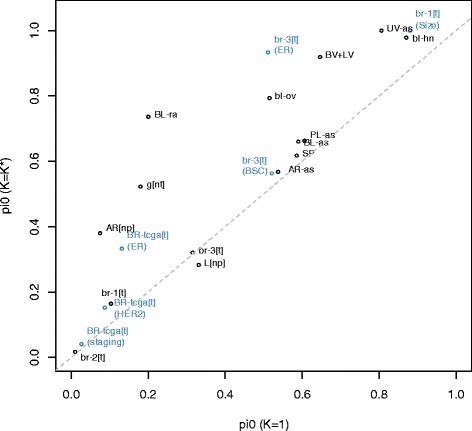
Comparison of Null Associations. Comparison of *π*_0_ (proportion of null association CpGs) from the *K* = 1 model with *π*_0_ from the *K* = K* model; only non-demographic variables are shown

We also considered the effect of **Ω** on CpG-specific associations of DNA methylation with **X**. As described below and in detail in Additional file 1: Section S5, we computed regression coefficients for logit-methylation (i.e. M-values) upon [**X**, **Ω**] for *K* ∈ {1, …, *K*_max_} (for *K* = 1 the covariate model was simply **X**); for **Ω** and for each covariate, we then used the resulting nominal p-values to estimate the proportion *π*_0_ of null associations. For demographic variables (age, sex, race), Additional file 1: Figure S5.1 illustrates the value of *π*_0_ for the overall association of **Ω** with DNA methylation ($K={K}^{*}= \max \left(2,\widehat{K}\right)$). For demographic variables, Additional file 1: Figure S5.2 provides a comparison of *π*_0_ from the *K* = 1 model with *π*_0_ from the *K* = K* model. Figure 4 displays a similar comparison for other variables. These figures demonstrate that adjustment by **Ω** very often resulted in higher values of *π*_0_, the estimated proportion of null associations. Exceptions were normal vs. cirrhotic liver (Fig. 4, Additional file 1: Table S5.2), age in *bl-hn* and sex in *AR[np]* and *AR-as* (Additional file 1: Figure S5.2, Table S5.1); for these variables, adjustment by **Ω** reduced *π*_0_. These cases may represent instances where **Ω** clarified single-locus associations; for example, adjusting for cellular heterogeneity in liver (e.g. hepatocytes vs. fibrous tissue) may clarify the detailed molecular profile of the two distinct types of liver pathologies (alcohol vs. viral cirrhosis). On the other hand, the proportion of null associations with **Ω** was typically low: except for the homogeneous tissue datasets *SP* and *BL+LV*, *π*_0_ was less than 0.2; of the others, except for the arsenic-exposure data sets *UV-as*, *AR-as*, and *PL-as*, *π*_0_ was extremely close to zero.

### Interpretation of putative cell types

**Fig. 5:**
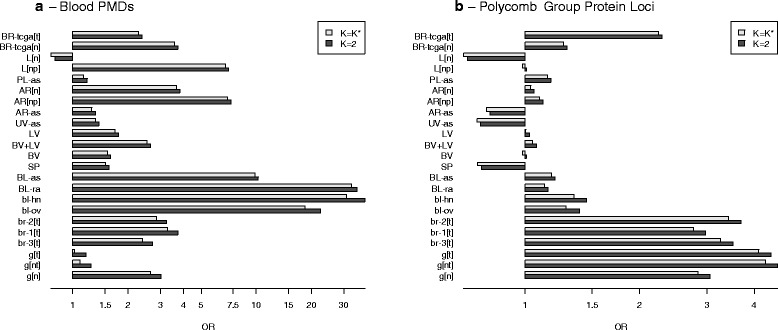
Gene-Set Analysis (DMPs and PcG Targets). Gene-set odds ratios, showing the association of gene set membership with the set of CpGs whose values are highly variable across fitted methylomes (*s*_*j*_^2^ > *q*_0.75_(*s* ^2^)). **a** Blood DMRs. **b** CpGs mapped to polycomb group protein genes

We examined the biological relevance of resulting matrices **M** in several different ways. First, for each data set, we computed row-variances *s*_*j*_^2^ (as described above) both for *K* = 2 and for ${K}^{*}= \max \left(2,\widehat{K}\right)$. For each of these two values of *K*, we classified each CpG *j* ∈ {1, …, *m*} by whether its row-variance *s*_*j*_^2^ lay above the 75th percentile *q*_0.75_(*s*^2^), reasoning that methylation of these loci vary more across cell types, and thus these CpGs could be important cell type discriminators. Next, we obtained a list of DMPs for differentiating distinct major types of leukocytes (*Blood DMPs*), and another list of CpGs mapped to genes considered Polycomb Group proteins (*PcG loci*), the construction of both lists described in detail in Additional file 1: Section S6. For each data set we computed the odds ratio for the association of high row-variance (defined as *s*_*j*_^2^ > *q*_0.75_(*s*^2^)) with DMP set membership (*Blood DMPs* or *PcG loci*), using Fisher’s exact test to compute the corresponding p-values. Odds ratios are depicted in Fig. 5, with log_10_ p-values given in Additional file 1: Table S6.1. *Blood DMP* status showed the highest associations in blood data sets, although somewhat high associations were also observed in *L[np]* and *AR[np]* (data sets having tissues with potentially inflammatory components to pathology). Data sets with tumors (*BR-tcga[t]*, *br-1[t]*, *br-2[t]*, *br-3[t]*, *g[nt]*, and *g[t]*) showed high association of *PcG loci* with cell-type distinguishing CpGs, but so did the data set with normal gastric tissue, *g[n]*. As shown in Additional file 1: Figure S6.1, the *Bilenky* DMPs, which were based on breast tissue, showed the highest association with cell-type discriminating CpGs in the data sets with breast tissue, although associations were also high in *L[np]*, *AR[n]*, and *AR[np]*. As shown in Additional file 1: Figure S6.2, the *REMC* DMPs, based on comparison of ectodermal/mesodermal/endodermal distinctions among embryonic stem cells, showed relatively weak (or negative) associations with cell-type distinguishing CpGs for all datasets. Figure 5 also suggests that odds ratios were slightly, though consistently, higher for *K* = 2 than for ${K}^{*}= \max \left(2,\widehat{K}\right)$, this may indicate that DMPs and polycomb targets are distinguished principally by two distinct types of cells, and that additional cell types add noise with respect to these specific comparisons.

**Fig. 6:**
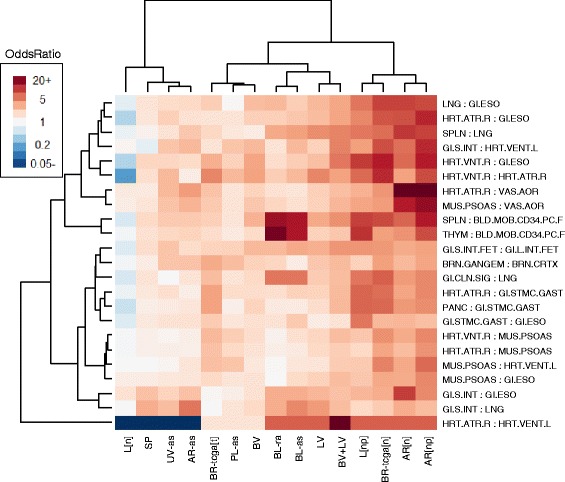
Gene-Set Analysis (Roadmap Epigenomics WGBS). Gene-set odds ratios for 450K data sets, showing association of sets of DMPs distinguishing various Roadmap Epigenomics WGBS specimens with the set of CpGs whose values are highly variable across fitted methylomes (*s*_*j*_^2^ > *q*_0.75_(*s* ^2^)). Clustering heatmap obtained from log-odds-ratios and using Ward’s method of clustering (“ward.D” in R *hclust* function)

We also developed a novel approach based on WGBS data from the Roadmap Epigenomics Project for 24 primary tissues. For each sample, we obtained the 470,909 CpGs overlapping with CpGs from either Infinium array (and having fewer than 3 missing values), clustering the tissue samples based on the 15,000 most variable of these CpGs (Manhattan distance metric with Ward’s method of clustering). The resulting dendrogram, shown in Additional file 1: Figure S7.1, demonstrates substantial clustering along general tissue type. We also applied our deconvolution algorithm to these 24 tissue samples (*K* = 6), with results shown in Additional file 1: Figure S7.2; note that the deconvolution of these tissues resulted in constituent cell types that roughly aligned with anticipated anatomical associations, e.g. tissues with substantial smooth or skeletal muscle mapped to one cell type, tissues with a substantial lymphoid/immune component mapped to another, and central nervous tissues map to yet another. We reasoned that *similar* tissue types would differ principally in the proportion of underlying normal constituent cell types, and thus provide information on cell-type heterogeneity underlying other tissues of similar type. Consequently, we selected the tissue pairs corresponding to the 25 smallest Manhattan distances (as calculated for the clustering in Additional file 1: Figure S7.1), with pairs illustrated as network edges in Additional file 1: Figure S7.3. Due to small numbers of DMPs (10 or fewer) we excluded two pairs; for each of the remaining 23 pairs, we identified, among the 15,000 CpGs most variable across all 24 tissue types, those CpGs that differed in methylation fraction by greater than 0.70 between the two samples; we considered these CpGs to be Infinium-specific DMPs for tissue-specific heterogeneity. Using these 23 sets of DMPs, we conducted a gene-set analysis as described in the previous paragraph. The clustering heatmap in Fig. 6 presents the odds ratios for the 450K data with ${K}^{*}= \max \left(2,\widehat{K}\right)$; the heatmap in Additional file 1: Figure S6.4 presents the odds ratios for the 27K data with ${K}^{*}= \max \left(2,\widehat{K}\right)$, and the odds ratios for *K* = 2 are given in Additional file 1: Figures S7.5 and S7.6. Corresponding p-values are given in Additional file 1: Tables S7.1, S7.2 and S7.3. Note that we excluded additional pairs from the 27K array analysis due to small DMP overlap with the 27K array. As shown in Fig. 6, positions that distinguished immune-related tissues (CD34+ hematopoietic stem cells vs. thymus or spleen) were highly associated with CpGs distinguishing cell types in the two 450K blood data sets, as well as in the mixed liver tissue dataset *L[np]* and the mixed arterial dataset *AR[np]*, consistent with the findings demonstrated in Fig. 5a. In the arterial data sets *AR[n]* and *AR[np]*, the normal breast data set *BR-tcga[n]* and to some extent the normal mixed vessel data set *BV+LV*, high associations were found for CpGs that distinguished smooth muscle content (aorta vs. psoas muscle, heart atrium vs. ventricle, heart atrium vs. esophagus). Interestingly, *AR[n]*, *AR[np]*, and *BR-tcga[n]* displayed associations with CpGs distinguishing lung and esophagus, potentially an epithelial cell comparison (although potentially also representing a distinction in smooth muscle content). All other positive associations were relatively weak. Strong negative associations with CpGs that distinguished right atrium from left ventricle were observed for *L[n]*, *SP*, *UV-as*, and *AR-as*, although these results may be driven by small numbers of CpGs (see p-values in Additional file 1: Table S7.2). Patterns were similar for *K* = 2 (Additional file 1: Figure S7.5). Patterns were similar in 27K blood data sets; additionally, the normal gastric data set g[n] displayed high association with DMPs distinguishing Roadmap stomach tissues (Additional file 1: Figure S7.4). Interestingly, *L[n]* was the only dataset displaying mostly negative (though weak) associations.

### Additional analyses

As described fully in Additional file 1: Section S8, we compared our proposed reference-free method with reference-based methods applied to the *BL-ra* and *BL-as* data sets, addressing (1) the extent to which the reference-based and reference-free approaches were consistent in their results; and (2) the extent to which our unsupervised approach may provide additional information on immune response and inflammation (as represented by distributions of leukocytes including their various activation states) beyond associations with simply the major types of leukocytes. Although unsupervised methods failed to return the exact biologically relevant cell types, they returned putative cell types that were related to the true types by a linear mixing matrix, and the phenotypic associations with true cell types were extremely consistent with associations produced in the reference free analysis, after accounting for the linear transformation (Additional file 1: Figures S8.3 through S8.6). Additionally, correlation of methylome **M** (from our reference-free method) with DMPs based on Roadmap WGBS data was substantially reduced after accounting for methylation differences among major types of leukocytes (Additional file 1: Figure S8.7 through Figure S8.10), suggesting that both reference-free and reference-based methylomes **M** contain similar cell-type information. Gene-set analyses demonstrated that both our approach and the reference-based method were similar in the immune sets they identified as functionally relevant to rheumatoid arthritis (Additional file 1: Figure S8.11), while our approach (with *K* = 7) identified several immune processes potentially functionally relevant to arsenic exposure but missed by the reference-based approach (Additional file 1: Figure S8.12).

We compared our method to Surrogate Variable Analysis (SVA [45]), a popular method currently used for reference-free analysis. With *K* = 7, SVA found many of the same functionally relevant immune processes, but they were evident in the residual associations, i.e. the CpG-specific associations obtained after adjusting for the SVA analogue of **Ω**, not in the surrogate variables that presumably represent cell mixture. However, after raising *K* to 33, the value estimated by SVA, results were more similar to those obtained by our method. This suggests that SVA produces results that are similar to our method, but using greater degrees-of-freedom.

Finally, as described in Additional file 1: Section S9, notable distinctions between normal and pathological tissues were revealed in an analysis of cell proportions **Ω** obtained using the Roadmap-derived methylomes as a reference (Additional file 1: Figures S9.1 through S9.3). In particular, gastric tumors differed from normal gastric tissues in having greater immunological/inflammation content but lesser gastrointestinal content, atherosclerotic carotid (and to some extent atherosclerotic aorta) differed from normal aorta in having greater immunological/inflammation content but lesser muscular content, and cirrhotic tissues differed from normal liver tissues in having greater immunological/inflammation content but lesser gastrointestinal content (with the pattern more striking for cirrhotic tissues related to viral infection than for cirrhotic tissues related to alcohol abuse).

### Simulations

In Additional file 1: Section S10 we describe simulations that illustrate the behavior of our proposed method. We compared our approach to that of ordinary nonnegative matrix factorization (NNMF) [46] as well as standard principal components analysis (PCA), which forms the fundamental basis of many existing reference-free algorithms (SVA [45], ISVA [32], RUV [21], RefFreeEWAS [27], ReFACTor [47]). By statistical criteria alone, PCA slightly outperformed both NNMF and our method, an anticipated result given that PCA solutions entail no constraints. However, our method provided mixing coefficients that were more interpretable biologically, as judged by the entropy of the mixing matrix that relates true cell proportions to their estimates. This improvement arises principally from the fact that PCA constrains constituent types to be orthogonal and typically produces z-scores as proxies for cell types, while our proposed method entails the precise constraints that arise naturally from the cell mixture problem. In addition, we demonstrated that our proposed method for estimating *K* compared favorably against several alternatives. We also demonstrated the difficulty of any unsupervised approach in estimating biologically interpretable cell types when the variation within the data set provides little information about those types: in particular, when the data do not support a distinction in cell type, the estimated number of classes may be smaller than the true number, and there may be consequent collapsing of categories. Finally, we present evidence that estimation of *K* is sensitive to the existence of cellular subtypes (e.g. immune activation states), and will tend to increase when there is substantial variation in distinct states of specific cell types. This phenomenon is consistent with the large value of *K* estimated for *BL-ra* (≥ 10 from Fig. 2b) and for the tumor data sets, as the histopathology of rheumatoid arthritis entails profound alterations in specific leukocytes [48, 49], while it is increasingly being recognized that cancer cells typically display extreme molecular heterogeneity [50].

## Discussion

We have proposed a simple method for reference-free deconvolution that provides both interpretable outputs, i.e. proportions of putative cell types defined by their underlying methylomes, as well as a method for evaluating the extent to which the underlying reflect specific types of cells. We have demonstrated these methods in a wide array of methylation datasets in various tissues and focused on differing exposures or outcomes.

Overall our deconvolution approach is similar to many others that have been proposed [29–33]. In particular, it is very similar to a recent publication that applied a convex-mixtures approach to deconvolve RNA expression [33]. Our approach differs from this one in that it deconvolves DNA methylation, with a corresponding constraint on the values of **M**. Our approach also differs from widely used PCA-based methods such as SVA in imposing biologically based constraints, thus resulting in mixture coefficients having greater biological interpretation and placing greater emphasis on coordinated cellular processes. In addition, importantly, we have provided a more comprehensive approach for interpreting the resulting columns of **M**, unlike most existing methods.

We have provided a novel approach for estimating the number $\widehat{K}$ of cell types, which we have shown to reflect the level of cellular heterogeneity anticipated from each tissue we analyzed. More heterogeneous tissues (blood, breast, and gastric tissues) resulted in higher values of $\widehat{K}$, while the more homogeneous tissues had lower values: $\widehat{K}=1$ for the (admittedly small) isolated lymphatic and blood vessel endothelium data sets, while $\widehat{K}=2$ for sperm and umbilical cord endothelial tissues. Note, however, that Fig. 2b reflects ambiguity in the choice of $\widehat{K}$ for sperm and equally supports the choice $\widehat{K}=1$; similar plots shown in Fig. 2b unambiguously suggest $\widehat{K}>1$ for two blood data sets and for an artery data set. A similar plot for UV-as (not shown) displayed an unambiguous preference for the choice of $\widehat{K}=2$, but the two putative types of cells did not associate with any metadata (Table 2). Taken together, these results demonstrate that our algorithm returns reasonably reliable values of $\widehat{K}$ reflecting cellular heterogeneity.

Cell mixture proportions **Ω** were typically significantly associated with major phenotypes of interest, with the notable exception of sperm, umbilical vein endothelium, and placental artery (the former two assumed to be homogenous tissues). Thus, the radically dimension-reduced DNA methylation data in **Ω** can still retain strongly significant associations with major phenotypes of interest. However, for other covariates, especially demographic confounders, there was considerable variation in significance, demonstrating that **Ω** can also show null associations with some covariates. Taken together, the results show that **Ω** can distinguish signal from noise. As the limma analysis demonstrated, residual signal can still exist in **Y** even after adjusting for **Ω**, although often in a more diminished capacity. In a few rare cases, the signal increased after adjusting for **Ω**. Taken together, these results suggest that a substantial proportion of the association between **Y** and phenotypic metadata **X** can be factored through the decomposition **Y** = **MΩ**^*T*^, occasionally clarifying the residual signal, but more often diminishing it. This finding is significant, as it would strongly suggest that the results of the vast majority of EWAS studies are driven by physiologic changes of the underlying composition of cells within the samples obtained. This is nicely highlighted by a recent report identifying a specific cell type driving the associations between smoking and changes in DNA methylation in peripheral blood [23]. This is in contrast to the prevailing current interpretation of most findings, which has aligned more strongly with the concept of metastable epialleles. These alleles represent loci where environmental conditions during development dictate ‘setpoints’ for the levels of methylation at particular gene sequences that are consistent across tissues within any person, also yielding differences in concordant gene expression [51–54]. The methods described in our work may have some utility for future discovery of these alleles in that within-person, cross-tissue comparisons of methylation profiles would be expected to be enriched for metastable alleles when the loci that are reflective of subsets of cell types are described and removed from comparisons.

On the other hand, we demonstrated that columns of **M** correlate with external biological annotation data in a manner concordant with their interpretation as methylomes specific to constituent cell types. *Blood DMP* status showed the highest associations in blood data sets, although also somewhat high associations in *L[np]* and *AR[np]*, data sets having tissues with potentially inflammatory components to pathology. Data sets with tumors (*BR-tcga[t]*, *br-1[t]*, *br-2[t]*, *br-3[t]*, *g[nt]*, and *g[t]*) demonstrated high associations with *PcG Loci*, reflecting the mitotic activity of tumors; that normal gastric tissue, *g[n]* also showed a high association with *PcG Loci* is consistent with the high level of cellular turnover in gastric tissues. The Bilenky DMRs showed the strongest associations for breast tissues, consistent with the fact that the Bilenky DMRs were obtained from breast tissue, but also demonstrated strong associations for liver pathologies, and in arterial tissues *AR[n]* and *AR[np]*. Breast and arterial tissues have a mix of epithelial and smooth muscle tissues, which may explain the arterial results. The association in *L[np]* may reflect the fibrous character of pathological liver tissue. REMC DMRs demonstrated only weak correlation or strong negative correlation with all tissues, perhaps reflecting the embryonic/developmental nature of the REMC DMRs.

Comparisons with DMPs constructed from Roadmap WGBS data also demonstrated that the columns of **M** reflect epigenetic content concordant with anatomical expectations; in particular, blood datasets displayed associations with DMPs distinguishing CD34+ hematopoietic stem cells vs. thymus or spleen, as were datasets *L[np]* and *AR[np]*, which both included tissue pathologies involving inflammation and immune response. Arterial data sets displayed associations with DMPs distinguishing smooth muscle from endothelium. Associations with Roadmap-based DMPs were typically weak for homogeneous tissues, in particular sperm. Interestingly, the normal liver tissue data set *L[n]* had mostly weak negative associations; one possible explanation is that the primary tissues available from the Roadmap were too dissimilar from normal liver tissue to distinguish subtle anatomical features. Using the Roadmap data as a pseudo-reference, normal and pathological tissues were revealed to differ anatomically along anticipated lines, specifically in that pathological tissues had greater cellular content reflective of immune or inflammation processes, and lesser gastrointestinal content (gastric and liver tissue) or muscular content (arterial tissue). Taken together, these results suggest that the columns of **M** reflect methylomes of constituent cell types.

We remark that the unsupervised deconvolution or approach we have proposed cannot be guaranteed to recover the methylomes of all constituent cell types; instead, it recovers the major axes of cellular variation. This was evident in the comparison of reference-based and reference-free deconvolution of blood datasets *BL-ra* and *BL-as*; for these datasets, the reference-free approach recovered the linear combination of reference methylomes most relevant to characterizing the underlying variation. However, when “re-mixed” back to proportions of known cell types using a reference methylome, associations with phenotypic metadata were consistent with those obtained from reference-based deconvolution. Functional implications were also typically similar; for example, supervised and unsupervised approaches identified several processes involving helper T-cells and T-cell polarization, consistent with known Th1/Th2 differentiation processes [49, 55] and T-Cell polarization processes [48, 56] involved in the etiology of rheumatoid arthritis. While on its surface this suggests that the reference-free approach has no value when a reference methylome is known (as is the case with blood), further analysis of the residual information in the unsupervised deconvolution demonstrated that reference-free deconvolution may occasionally reveal distinctions in cell type relevant to characterizing the underlying variation in the dataset but more subtle than the potential distinctions fixed in advance by the reference set; this was evident in the *BL-as* analysis, for which the unsupervised approach identified *Regulators of T-Cell Activation* and *T-Cell Proliferation* as significant processes potentially altered by arsenic exposure in Bangladesh. In fact, the impact of arsenic exposure on regulation of T-cells has been noted [57], and even observed in another study conducted in Bangladesh [58]. Thus, the reference-free approach may provide important information that complements a reference-based approach.

We remark that the failure to retrieve the exact biologically relevant types is a feature common to all unsupervised methods, which (as we demonstrate by simulation) can differentiate heterogeneity only along the major axes of variation within a data set. Comparisons with other existing approaches, both in data analysis as well as simulations, suggest that our proposed method produces results that are similar with other unsupervised methods in their ultimate functional conclusions. However, most existing methods for deconvolving DNA methylation data rely on adaptations of PCA, thus producing orthogonal surrogates of cellular function having no clear cell-type interpretation. Additionally, we provide some evidence that PCA-based approaches may require larger degrees-of-freedom to capture the functional relevance of phenotypic associations (conceived as coordinated cellular processes rather than disparate unrelated effects), although our results are by no means conclusive. However, an attractive feature of our proposed method is a likelihood-based method of estimating the underlying dimensionality of the cell mixture component, motivated by biological constraints instead of an ad-hoc PCA-based model.

As a general point, we have demonstrated the links suggested in Fig. 1; thus, we have shown that it is possible to use a reference-free approach to characterize the extent to which phenotypic associations with DNA methylation data are explained by differences in constituent cell types. We remark that such distinctions may be subtle, such as variation in smooth muscle content or the presence of leukocytes with specialized immunological states. There may still exist associations residual to those with variations in putative underlying cell types, although they will often be diminished after adjusting for cell type in the manner we have proposed. Other reference-free approaches can also distinguish between associations driven by variation in cell type and those that are more focal to individual CpG sites, but our proposed method is based on a biologically based model that emphasizes the coordination of cellular processes, and leads naturally to a bootstrap-based method for estimating *K*. Additionally, we have proposed a method for interpreting the cell-mixture component of variation in DNA methylation data sets using the estimated methylome matrix, which presumably reflects coordinated biological activity. While similar insights may be obtained simply by examining the CpG-specific associations, we note that there is ongoing controversy on what “adjustment for cell-type” means in the context of EWAS analysis. We have previously argued that all epigenetic variation is ultimately mediated by cell-type, if the meaning of “cell-type” is conceived of broadly enough [20]; a more useful framing of the question is how to identify types of cells that are relevant to the biological variation being studied. Our proposed approach helps in partitioning the underlying variation into units that resemble cell-specific methylomes, so that these methylomes or the overt functional characteristics of these cells may be further analyzed using additional biological characterization data. Thus, our approach emphasizes the extent to which biological processes are coordinated at the cellular level.

We remark on a few current limitations of our approach. One is that we have used a crude gene-set procedure based on variance, which removes “signed” information and thus precludes the use of algorithms based on expression signature, such as CTen [59]. Another related limitation is a lack of relevant annotation data. Further work is necessary to adapt the method we have proposed here to “signed” comparisons, thus enabling a wider array of annotation tools, and to develop other relevant annotation datasets relevant to identifying subtle cell types.

## Conclusions

We have proposed a simple method for reference-free deconvolution that provides both interpretable outputs, i.e. proportions of putative cell types defined by their underlying methylomes, as well as a method for evaluating the extent to which the underlying reflect specific types of cells. We have demonstrated these methods in a wide array of methylation datasets in various tissues and focused on differing exposures or outcomes. Our methodology permits an explicit quantitation of the mediation of phenotypic associations with DNA methylation by cell composition effects. Although more work is needed to investigate functional information related to estimated methylomes, our proposed method provides a novel and useful foundation for conducting DNA methylation studies on heterogeneous tissues lacking reference data.

## Methods

### Matrix factorization with convex constraints

To obtain a biologically meaningful deconvolution, we assume an *m* × *n* matrix **Y** representing methylation data collected for *n* subjects or specimens, each measured on an array of *m* CpG loci, and that the measured values are constrained to the unit interval [0, 1]. We assume the following decomposition: **Y** = **MΩ**^*T*^, where **M** is a *unknown m* × *K* matrix representing *m* CpG-specific methylation states for each of *K* cell types (with row vectors representing profiles each individual CpG) and **Ω** is an *unknown n* × *K* matrix representing subject-specific cell-type distributions (each row representing the cell-type proportions for a given subject, i.e. the entries of **Ω** lie within [0, 1] and the rows of **Ω** sum to values less than one). Additional file 1: Section S1 describes estimation details, which involve alternating the estimation of **Ω** and of **M** using applications of quadratic programming [60]. Additional file 1: Section S2 describes a method of estimating *K* by generating bootstrap samples, obtaining their deconvolution for each assumed value of *K*, and assessing their fit on the samples left out of the bootstrap.

### Empirical examination of proposed methods

We removed chromosome Y data from all datasets; and we also removed chromosome X data from all but the breast datasets. For the 450K data sets downloaded from TCGA, and for the 450K data collected to investigate associations with arsenic exposure in Bangladeshi neonates [3, 9], we used the *FunNorm* algorithm (*Bioconductor* package *minfi*) to process the raw *idat* files; we obtained all other data sets as processed average beta values from Gene Expression Omnibus (GEO). For 450K data sets, we excluded CpGs with cross-hybridizing probes or probes with SNPs [61], and used the *BMIQ* algorithm [62] (*Bioconductor* package *wateRmelon*) to align the scales of Type I and Type II probes. Finally, for each data set, we excluded CpGs having missing measurements for over half the specimens.

### Associations with phenotypic metadata

As described in Additional file 1: Section S4, permutation tests were used to assess omnibus significance of covariates **X** with fitted cell proportions **Ω**. As described in Additional file 1: Section S5, we further compared associations of **Y** with **X** before and after including terms from **Ω** in the regression model for **Y**, using the *limma* procedure [63] (via the R package *limma*) to compute regression coefficients, using the R package *qvalue* to estimate both q-values and the overall proportion *π*_0_ of null associations.

### Interpretation of putative cell types

We obtained a list of DMPs for differentiating distinct major types of leukocytes (*Blood DMPs*) from the Reinius reference set [25], and constructed a set of CpGs mapped to genes considered Polycomb Group proteins (*PcG loci*), compiled from four references [64–67] as in our previous articles [20, 27]. We also constructed a set of CpGs based on differentially methylated regions (DMRs) obtained from WGBS data collected by the Epigenomics Roadmap Project. Additional file 1: Section S6 describes the details of the construction of these DMP sets. In addition, we developed a novel approach based on WGBS data from the Roadmap Epigenomics Project for 24 primary tissues, described in detail in Additional file 1: Section S7. WGBS data were aligned with 450K data using the new *methyLiftover* software.

### Additional analyses on 450K blood datasets

To compare reference-based analysis with our proposed approach, we analyzed the two 450K blood data sets, *BL-ra* and *BL-as*, estimating for each data set two sets of cell-type proportion matrices (*K* = 7): **Ω**_0_ (reference-based) and **Ω**_1_ (reference-free). Details appear in Additional file 1: Section S8. Briefly, to obtain the mixing matrix **Ψ** that relates matrices **M**_0_ and **M**_1_, we used a constrained projection similar to that used to obtain the reference-based cell proportion matrix **Ω**_0_, and compared **M**_1_ with **M**_1_ − **M**_0_**Ψ**^*T*^ by identifying CpGs with high variation across their constituent methylomes. In addition, we compared these highly varying CpGs with with immune activation and immune regulation pathways compiled from six sources [68–74].

### Additional analyses on datasets with normal and pathological tissue

We projected Infinium data from each of the three datasets sets *g[nt]*, *AR[np]*, and *L[np]* onto the profile matrix **M** obtained by decomposing the Roadmap WGBS data (**Y** = **MΩ**^*T*^); we then averaged the resulting specimen-specific cell proportions **Ω** over tissue status (normal gastric tissue vs. gastric tumor, normal aorta vs. atherosclerotic aorta and atherosclerotic carotid, and normal liver vs. alcohol-related cirrhotic liver and cirrhotic liver due to viral infection). Details and results appear in Additional file 1: Section S9.

### Simulations

We based our simulations on estimates obtained from the *BL-ra* data set. A Dirichlet distribution was used to model reference-based estimates of cell composition from the *BL-ra* data set, and was also used to generate “true” values of cell proportion. Additional microarray error was incorporated using beta distributions. We used various measures of statistical fit and coefficient interpretability to compare our method with PCA and NNMF, and compared our proposed method of estimating *K* with a previously proposed method based on random matrix theory [32], as well as an ad-hoc procedure commonly used in factor analysis. Details appear in Additional file 1: Section S10.

## Abbreviations

27K, HumanMethylation27; 450K, HumanMethylation450; CpG, Cytosine-phosphate-Guanine; DMP, differentially methylated position; EWAS, Epigenomewide Association Study; NNMF, nonnegative matrix factorization; PCA, principal components analysis; PcG, Polycomb group; SVA, Surrogate Variable Analysis; TCGS, The Cancer Genome Atlas; WGBS, whole genome bisulfite sequencing.

## Additional files

Additional file 1: Reference-free deconvolution of DNA methylation data and mediation by cell composition effects – Supplementary Information. This file contains supplementary figures, tables, and detailed descriptions of methods and results. (DOCX 1733 kb) Additional file 2: Supplementary Information Section S5 P-Value Histograms. This file contains histograms of p-values from limma analyses. (ZIP 39 kb) Additional file 3: Supplementary Information Section S5 P-Value Trajectories. This file contains illustrations of the trajectory of *π*_0_ (proportion of null associations in limma analyses) as *K* varies across different values. (ZIP 82 kb)
