# Supplementary material for: Reference-free deconvolution of DNA methylation data and mediation by cell composition effects
Source: BMC Bioinformatics. 2016 Jun 29;17:259. doi: 10.1186/s12859-016-1140-4 (PMC4928286; doi:10.1186/s12859-016-1140-4)
Supplement: Additional file 1: — Reference-free deconvolution of DNA methylation data and mediation by cell composition effects – Supplementary Information. This file contains supplementary figures, tables, and detailed descriptions of methods and results. (DOCX 1733 kb) [file 12859_2016_1140_MOESM1_ESM.docx]

**Reference-free deconvolution of DNA methylation data and mediation by cell composition effects – Supplementary Information**

E. Andres Houseman^1^, Molly Kile^1^, David C. Christiani^2^, Tan A. Ince^3^, Karl T. Kelsey^4^, Carmen J. Marsit^5^

1. School of Biological and Population Health Sciences, College of Public Health and Human Sciences, Oregon State University; Corvallis, OR, USA. Email: [andres.houseman@oregonstate.edu](mailto:andres.houseman@oregonstate.edu)

2. Department of Environmental Health, Harvard T. H. Chan School of Public Health; Boston, MA, USA.

3. Department of Pathology, University of Miami, Miller School of Medicine; Miami, FL, USA.

4. Department of Epidemiology, Department of Pathology and Laboratory Medicine, Brown University

5. Department of Community and Family Medicine, Dartmouth Medical School; Hanover, NH, USA.

**Section S1 – Convex Deconvolution of DNA Methylation Data**

We assume an $m\times n$ matrix $\mathbf{Y}$ representing methylation data collected for $n$ subjects or specimens, each measured on an array of $m$CpG loci, and that the measured values are constrained to the unit interval $[0,1]$. We explicitly write $\mathbf{Y}$ in terms of its row vectors $\mathbf{Y}=(\mathbf{y}_{j}^{(r)\mathrm{T}})_{j\in\{1,...,m\}}^{\mathrm{T}}$ and its column vectors $\mathbf{Y}=(\mathbf{y}_{i}^{(c)})_{i\in\{1,...,n\}}$. We also assume the following decomposition: $\mathbf{Y}=\mathbf{M\Omega}^{\mathrm{T}}$, where $\mathbf{M}=(\mathbf{\mu}_{j}^{\mathrm{T}})_{j\in\{1,...,m\}}^{\mathrm{T}}=(\mu_{jk})_{j\in\{1,...,m\},k\in\{1,...,K\}}$ is a *unknown* $m\times K$ matrix representing $m$ CpG-specific methylation states for each of $K$ cell types (with row vectors representing profiles each individual CpG) and $\mathbf{\Omega}=(\mathbf{\omega}_{i}^{\mathrm{T}})_{i\in\{1,...,n\}}^{\mathrm{T}}=(\omega_{ik})_{i\in\{1,...,n\},k\in\{1,...,K\}}$ is an *unknown* $n\times K$ matrix representing subject-specific cell-type distributions (each row representing the cell-type proportions for a given subject, i.e. the entries of $\mathbf{\Omega}$ lie within $[0,1]$ and the rows of $\mathbf{\Omega}$ sum to values less than one). For a fixed number $K$ of assumed cell types, we estimate $\mathbf{M}$ and $\mathbf{\Omega}$ as follows:

1. Start with an initial estimate of $\mathbf{M}$.
2. Fixing $\mathbf{M}$, construct a new $\mathbf{\Omega}=(\mathbf{\omega}_{i}^{\mathrm{T}})_{i\in\{1,...,n\}}^{\mathrm{T}}$: for each $i\in\{1,...,n\}$, minimize $\left\|\mathbf{y}_{i}^{(c)}-\mathbf{M\omega}_{i}\right\|^{2}$ subject to the constraints $0\leq\omega_{ik}\leq1$ and $\sum_{k=1}^{K} \omega_{ik}\leq1$.
3. Fixing $\mathbf{\Omega}$, construct a new $\mathbf{M}=(\mathbf{\mu}_{j}^{\mathrm{T}})_{j\in\{1,...,m\}}^{\mathrm{T}}$: for each $j\in\{1,...,m\}$, minimize $\left\|\mathbf{y}_{j}^{(r)}-\mathbf{\Omega\mu}_{j}\right\|^{2}$ subject to the constraints $0\leq\mu_{jk}\leq1$.
4. Repeat steps (1)-(2) a specific number of times.

The constrained optimizations in steps (1) and (2) can easily be achieved using a quadratic programming algorithm[^1^](#_ENREF_1) implemented in the *R* library *quadprog*. We note that if $\mathbf{M}$ is chosen reasonably well, a relatively few number of iterations will be necessary to achieve near-convergence. For the present analysis, 25 iterations were used; Figure S2.1 displays box-and-whisker plots for the distribution of absolute differences (absolute values of the entries of $\mathbf{Y}-\mathbf{M\Omega}^{\mathrm{T}}$) between the last two iterations of the $K=2$ fit, while Figure S2.2 displays the corresponding plot for $K=K^{*}=\max(2,\hat{K})$, where $\hat{K}$ was the estimated number of classes as described below in Section S3. As suggested by the figures, the error was typically less than 0.01, and often about 0.001 or less.

For the present analysis, we have initialized $\mathbf{M}$ step (0) as follows: we used hierarchical clustering to cluster the columns of$\mathbf{Y}$ (i.e. using a Manhattan metric and Ward’s method of clustering), formed $K$ classes from the resulting dendrogram, and initialized $\mathbf{M}$ as the $K$ mean methylation vectors corresponding to each class. In this way, $\mathbf{M}$ was initialized in a manner consistent with the RPMM algorithm[^2^](#_ENREF_2), widely used in DNA methylation analysis.

A substantial portion of the variation between cell-type specific methylomes will be driven only by the most evidently variable CpG loci, with the remaining loci contributing only noise; consequently, for the present analysis we have selected the$m=5000$ most variable CpGs (within each data set) for the 27K data sets, and the $m=10,000$ most variable CpGs (within each data set) for the 450K data sets. However, subsequent to step (3) in the algorithm, with the value of $\mathbf{\Omega}$ estimated, we constructed a new $\mathbf{M}$ for the full array, as in step (2).

| Figure S1.1 – Convergence Error, *K*=2 | Figure S1.1 – Convergence Error, *K*=*K** |
| --- | --- |

**Section S2 – Bootstrap Method for Determining the Number of Classes *K***

Because conventional model-fitting statistics such as AIC and BIC fail in high-dimensional problems, where the number of model parameters greatly exceeds the sample size, we used a bootstrap technique to estimate the optimal number of classes, $\hat{K}$. For each data set, we sampled the specimens with replacement *R*=1000 times; for each bootstrap sample $r$ and for $2\leq K\leq K_{\max}$ we fit the model described above in Section S2 to obtain estimates $\mathbf{M}^{(r)}$ and $\mathbf{\Omega}^{(r)}$(note that we used only the most variable CpG loci, as described in Section S2). Due to the large number of resampling iterations, we iterated the algorithm of Section S2 only 10 times instead of 25. Each bootstrap sample omits approximately 36.8% of the data, so with each remaining “out-of-bag” data set $\mathbf{Y}^{(-r)}=(y_{ji}^{(-r)})_{i\in\{1,...,N^{(-r)}\},j\in\{1,...,m\}}$, of sample size $N^{(-r)}\approx0.368 n$, we constructed deviance statistics as follows. For $K=1$ (no mixture), the bootstrap deviance was calculated as

$D_{1}^{(r)}=\frac{1}{N^{(-r)}}\sum_{j=1}^{m} \left\{n_{j}^{(-r)}\log[2\pi\sigma_{1,j}^{2(r)}]+[\sigma_{1,j}^{2(r)}]^{-1}\sum_{i=1}^{N^{(-r)}} (y_{ji}^{(-r)}-\mu_{j}^{(r)})^{2}\right\}$,

where $\mu_{j}^{(r)}$ and $\sigma_{j}^{2(r)}$ were, respectively, the mean and variance calculated for CpG locus $j$ from the bootstrap sample, and $n_{j}^{(-r)}\leq N^{(-r)}$ was the out-of-bag sample size available for that locus (i.e. excluding missing values); note that the variance was calculated using the maximum likelihood approach, $\sigma_{1,j}^{2(r)}=[n_{j}^{(r)}]^{-1}\sum_{i=1}^{n_{j}^{(r)}} (y_{ji}^{(r)}-\mu_{j}^{(r)})^{2}$ (with $n_{j}^{(r)}$denoting CpG-specific bootstrap sample size and $y_{ji}^{(r)}$ a bootstrapped value), rather than the more common restricted maximum likelihood method with denominator $n_{j}^{(r)}-1$. For $K\geq2$, the out-of-bag data $\mathbf{Y}^{(-r)}$ and the bootstrap estimate $\mathbf{M}^{(r)}=(\mathbf{\mu}_{j}^{(r)\mathrm{T}})_{j\in\{1,...,m\}}^{\mathrm{T}}$ were used to obtain an out-of-bag estimate of cell mixture proportions $\mathbf{\Omega}^{(-r)}=(\mathbf{\omega}_{i}^{(-r)\mathrm{T}})_{i\in\{1,...,N^{(-r)}\}}^{\mathrm{T}}$, as in step (1) of the algorithm described in Section S2. The bootstrap deviance for $K\geq2$ was then calculated as

$D_{K}^{(r)}=\frac{1}{N^{(-r)}}\sum_{j=1}^{m} \left\{n_{j}^{(-r)}\log[2\pi\sigma_{K,j}^{2(r)}]+[\sigma_{K,j}^{2(r)}]^{-1}\sum_{i=1}^{N^{(-r)}} (y_{ji}^{(-r)}-\mathbf{\mu}_{j}^{(r)\mathrm{T}}\mathbf{\omega}_{i}^{(-r)})^{2}\right\}$,

where $\sigma_{K,j}^{2(r)}=[n_{j}^{(r)}]^{-1}\sum_{i=1}^{n_{j}^{(r)}} (y_{ji}^{(r)}-\mathbf{\mu}_{j}^{(r)\mathrm{T}}\mathbf{\omega}_{i}^{(r)})^{2}$was calculated from the bootstrap sample, in a manner similar to $\sigma_{1,j}^{2(r)}$.

For $1\leq K\leq K_{\max}$, we summarized the deviance statistics $\{D_{K}^{(1)},...,D_{K}^{(1000)}\}$ by mean, median, and trimmed mean (trimming the upper and lower quartiles). We chose $\hat{K}$ as the value of $K$ that minimized the trimmed mean bootstrap deviance.

**Section S3 – Descriptive Overview of Data Sets**

Figure S1.1 shows the clustering of the 23 data sets used in this analysis, based on Ward’s method of clustering (specifically, the *Ward.D* implementation of R version 3.2.2) applied to Manhattan distances computed on mean methylation profiles on 26,476 CpG sites common to all 23 data sets. Figure S1.2 depicts the number of CpG sites used in subsequent analysis for each data set, with the proportion of observed data for each CpG indicated by color (note that for each data set, the majority of CpGs analyzed were observed for 100% of specimens in the data set).

We remark on the conventions we used for constructing short codes used to identify each data set: initial letters are lower-case for 27K data sets, upper-case for 450K data sets; for datasets consisting of mixed normal and pathological tissues and broken into subsets, the brackets indicate whether the data set contains normal [n], tumor [t], or other non-tumor pathological data [p].

| Figure S1.1 – Clustering of Infinium 27K and 450K Data Sets | Figure S1.2 – Summary of Number of CpGs and Observed Data |
| --- | --- |

**Section S4 – Permutation Test for Determining Associations with Metadata across Classes**

The row-vectors $(\mathbf{\omega}_{i}^{\mathrm{T}})_{i\in\{1,...,n\}}^{\mathrm{T}}=(\omega_{ik})_{i\in\{1,...,n\},k\in\{1,...,K\}}$ (as defined above in Section S2) are approximately Dirichlet distributed, but a less computationally method of modeling their associations with phenotypic metadata is to use quasi-likelihood. Specifically, for an $n\times d$ covariate matrix $\mathbf{X}=(\mathbf{x}_{i}^{\mathrm{T}})_{i\in\{1,...,n\}}^{\mathrm{T}}$, we propose the following approximate model:

$\mathrm{E}(\omega_{ik})=\theta_{ik}=\mathrm{logit}^{-1}(\mathbf{\gamma}_{k}^{\mathrm{T}}\mathbf{x}_{i})$, $\mathrm{var}(\omega_{ik})\propto\theta_{ik}(1-\theta_{ik})$,

where $\mathbf{\gamma}_{k}$ is a $d\times1$ vector of parameters for cell type $k$. This model can easily be fit using the *R* function *glm* with family set to *quasibinomial*. For each $k\in\{1,...,K\}$, *glm* will supply a vector of nominal p-values $\mathbf{p}_{k}=(p_{k1},...,p_{kd})$ corresponding to the coefficient estimates $\hat{\mathbf{\gamma}}_{k}$. If $C\subset\{1,...,d\}$ corresponds to a specific set of coefficients to be tested, then $p_{K}^{(C)}=\min\{p_{kl}:l\in C,k\in\{1,...,K\}\}$ measures the strength of evidence for an association of $\mathbf{\omega}_{i}$ with the phenotype represented by $C$. Correspondingly, $p_{\bullet}^{(C)}=\min\{p_{K}^{(C)}:K\in\{2,...,K_{\max}\}\}$ represents the strength of evidence for the association of the phenotype with cell-type under any assumed number of classes, and is thus agnostic in the choice of $K$. The null distribution of $p_{K}^{(C)}$ or $p_{\bullet}^{(C)}$ can easily be generated by permuation as follows. For one permutation iteration $r\in\{1,...,R\}$, permute the rows of the submatrix of $\mathbf{X}$ corresponding to the members of $C$, fit the model above, and generate the minimum p-value test statistics $p_{K}^{(C)(r)}$ and $p_{\bullet}^{(C)(r)}$; the model-specific p-value for an assumed number of classes $K$ is then $p_{K}^{(C)*}=R^{-1}\sum_{r=1}^{R} 1\left[p_{K}^{(C)(r)}\leq p_{K}^{(C)}\right]$, and the omnibus p-value over all assumed values of $K$ is $p_{\bullet}^{(C)*}=R^{-1}\sum_{r=1}^{R} 1\left[p_{\bullet}^{(C)(r)}\leq p_{\bullet}^{(C)}\right]$. Table 2 of the main text provides the omnibus p-values $p_{\bullet}^{(C)*}$ for each set of covariates considered for each data set we analyzed ($R=1000$ permutations). Supplementary file *Houseman-DNAm-deconvoluton-Supplement-S4-plots.pptx* illustrates the progression of p-values $p_{k}^{(C)*}$ as $K$ varies across different values; the file also provides clustering heatmaps illustrating the relationships between each covariate and $\mathbf{\omega}_{i}$, for $K=K^{*}=\max(2,\hat{K})$.

**Section S5 – Analysis of CpG-Specific Associations via Limma**

In order to assess the impact of the data reduction implied by the decomposition $\mathbf{Y}=\mathbf{M\Omega}^{\mathrm{T}}$ on CpG-specific associations, we used the *limma* procedure[^3^](#_ENREF_3) in the *R*/*Bioconductor* library *limma* to model CpG specific associations $\mathrm{E}[\tilde{\mathbf{y}}_{j}^{(r)}]=\mathbf{\Omega\alpha}+\mathbf{X\beta}$, where $\tilde{\mathbf{y}}_{j}^{(r)}=[\mathrm{logit}(y_{ji})]_{i\in\{1,...,n\}}$ denotes the row-vector of logit-transformed beta values (i.e. the *M*-values often used for CpG-specific analysis), $\mathbf{\alpha}$ is a $K\times1$ vector of covariates on $\mathbf{\Omega}$ (for a presumed value of $K$), and $\mathbf{\beta}$ is a $d\times1$ vector of covariates on $\mathbf{X}$. For each coviariate represented by a single regression coefficient, we captured the nominal CpG-specific p-values reported by the procedure; for covariates represented by multiple coefficients (e.g. categorical covariates) we formed the appropriate F-statistic using the relevant data elements returned by the procedure, subsequently calculating the corresponding CpG-specific p-values. Using the *R*/*Bioconductor* library *qvalue*, we transformed each resulting set of p-values to q-values, specifically estimating the proportion $\pi_{0}$ of null associations. For demographic variables (age, sex, race), Figure S5.1 provides a comparison of $\pi_{0}$ from the $K=1$ model (i.e. omitting the $\mathbf{\Omega\alpha}$ term) with $\pi_{0}$ from the $K^{*}=\max(2,\hat{K})$. Figure 4 of the main text provides the same comparison for other variables. Additionally, the supplementary compressed folder *Supplement-S5-histograms.zip* contains illustrations of p-value histograms, while the supplementary compressed folder *Supplement-S5-p-value-plots.zip* contains illustrations of the trajectory of $\pi_{0}$ as $K$ varies across different values. Tables S5.1 and S5.2 below tabulate the $\pi_{0}$ values shown in Figures 4 and S5.2. For each phenotype, we also used a paired Wilcoxon test to compared log-q-values computed with $K=1$ to those computed with $K=K^{*}$; all p-values were < 10^-16^, except for histology in *br-3[t]* (p=2.1 x 10^-16^).

| Figure S5.1 – Estimates of $\pi_{0}$ for $\mathbf{\Omega}$ with $K^{*}=\max(2,\hat{K})$ | Figure S5.2 – Comparison of Estimated $\pi_{0}$ for Demographic Variables |
| --- | --- |

| Table S5.1 – Null probabilities $\pi_{0}$ for Age, Sex, and Race coefficients    All paired Wilcoxon p-values < 10^-16^ | Table S5.2 – Null probabilities $\pi_{0}$ for Disease and Exposure Phenotypes    All paired Wilcoxon p-values were < 10^-16^, except for histology in *br-3[t]* (p=2.1 x 10^-16^) |
| --- | --- |

**Section S6 – Interpretation of Putative Cell Types Using Basic Annotation Data**

We examined the biological relevance of $\mathbf{M}$ in several different ways. First, for each data set, we computed row-variances $s_{j}^{2}$ both for $K=2$ and for $K^{*}=\max(2,\hat{K})$. For each of these two values of $K$, we classified each CpG $j\in\{1,...,m\}$ by whether its row-variance $s_{j}^{2}$ lay above the 75^th^ percentile $q_{0.75}(s^{2})$ for the data set and choice of $K$. Next, we obtained a list of DMPs for differentiating distinct major types of leukocytes (*Blood DMPs*) from the Reinius reference set[^4^](#_ENREF_4). Specifically, we used the *limma* procedure[^3^](#_ENREF_3) to fit a linear model for DNA methylation (average betas obtained via BMIQ normalization[^5^](#_ENREF_5) of data obtained from GEO, Accession # GSE35069) with a 10-level categorical variable representing the 10 categories of cell types assayed in the data set (reference level = *Whole Blood*). From the results, we constructed for each CpG an F-statistic representing the ability of the CpG to distinguish leukocyte lineages, using the following 6-degree-of-freedom contrast matrix (motivated by an interest in distinguishing successively fine lineages):

.

CpGs were sorted by the resulting F-statistics. CpGs corresponding to the 1000 largest were used as DMPs for tests involving 27K array data, while CpGs corresponding to the10,000 largest were used for tests involving the 450K array data.

We also constructed another set of CpGs, those mapped to genes considered Polycomb Group proteins (*PcG loci*), compiled from four references[^6-9^](#_ENREF_6) as in our previous article[^10^](#_ENREF_10).

We constructed another set of CpGs based on differentially methylated regions (DMRs) obtained from WGBS data collected by the Epigenomics Roadmap Project. Data were downloaded September 29, 2015 from http://egg2.wustl.edu/roadmap/data/byDataType/dnamethylation. The *Bilenky* set (DMRs/mbilenky_DMRs.xlsx), based on breast tissue data, differentiates luminal from myoepithelial cells. The *REMC* set (DMRs/REMC_DMRs_corrected.xlsx), based on embryonic stem cell data, provides DMRs for differentiating endodermal, mesodermal, and ectodermal tissues. While DMRs are provided separately for each tissue type, we are not able to make signed comparisons with our method, so we combined DMRs for the three types of embryonic cells and for the two types of breast tissues. Infinium-specific DMPs for each of these sets were determined by calculating the intersection of each DMR with CpGs available on the Infinium arrays. Note that the 450K array positions as well as the WGBS positions are given in *hg19* coordinates, but the 27K array positions are given in *hg18* coordinates; thus, for the CpGs on the 27K array but excluded from the 450K array, we used *UCSC Genomes Browser* to determine their *hg19* coordinates.

For each data set we computed the odds ratio for the association of high row-variance, $s_{j}^{2}>q_{0.75}(s^{2})$, with DMP set membership (*Blood DMPs*, *PcG loci*, *Bilenky DMPs*, and *REMC DMPs*), using Fisher’s exact test to compute the corresponding p-values. Odds ratios for *Blood DMPs* and *PcG loci* are depicted in Figure 5, with log_10_ p-values given in Table S6.1. Odds ratios for *Bilenky DMPs* and *REMC DMPs* are depicted in Figures S6.1 and S6.2, with log_10_ p-values given in Table S6.1.

Note that the CpGs having high row-variance in $\mathbf{Y}$ were not identical with those having high row-variance in $\mathbf{M}$. Table S6.x displays the log-odds ratios for the association of high row-variance in $\mathbf{Y}$ (i.e. whether the CpG was used for the initial decomposition as in Section S2) with high row-variance ($s_{j}^{2}>q_{0.75}(s^{2})$) in $\mathbf{M}$(fit with$K=K^{*}=\max(2,\hat{K})$), typically quite high as anticipated; however, Table S6.x also displays the percentage of CpGs with discordant status, typically 10-20%.

Table S6.1 – P-values for Gene Set Analysis of Basic Annotation Data (Negative Base-10 Logarithmic Scale)

Table S6.2 – Association of High Row Variance Status between $\mathbf{Y}$ and $\mathbf{M}$

| Figure S6.1 – Odds Ratios for Bilenky DMPs | Figure S6.2- Odds Ratios for REMC DMPs |
| --- | --- |

**Section S7 – Interpretation of Putative Cell Types Using Roadmap Epigenomics WGBS Data**

We developed a novel gene-set approach based on WGBS data from the Roadmap Epigenomics Project for 24 primary tissues. For each sample, we obtained the 470,909 CpGs overlapping with CpGs from either Infinium array (similar to the manner described in Section S6) and having fewer than 3 missing values. We clustered the tissue samples based on the 15,000 most variable of these CpGs (Manhattan distance metric with Ward’s method of clustering, specifically, the *Ward.D* implementation of *R* version 3.2.2). The resulting dendrogram, shown in Figure S7.1, demonstrates substantial clustering along general tissue type. We also applied our deconvolution algorithm (Section S2) to these 24 tissue samples ($K=6$), with results shown in Figure S7.2; note that the deconvolution of these tissues resulted in constituent cell types that roughly aligned with anticipated anatomical associations, e.g. tissues with substantial smooth or skeletal muscle map to one cell type, tissues with a substantial lymphoid/immune component mapped to another, and central nervous tissues mapped to yet another. We reasoned that *similar* tissue types would differ principally in the proportion of underlying normal constituent cell types, and thus provide information on cell-type heterogeneity underlying other tissues of similar type. Consequently, we selected the tissue pairs corresponding to the 25 smallest Manhattan distances (as calculated for the clustering in Figure S7.1), with pairs illustrated as network edges in Figure S7.3. Due to small numbers of DMPs (10 or fewer) we excluded two pairs (left vs. right ventricles of the heart and small intestine vs. sigmoid colon); for each of the remaining 23 pairs, we identified, among the 15,000 CpGs most variable across all 24 tissue types, those CpGs that differed in methylation fraction by greater than 0.70 between the two samples; we considered these CpGs as Infinium-specific DMPs for tissue-specific heterogeneity. Using these 23 sets of DMPs, we conducted a gene-set analysis as described in Section S6. The clustering heatmap in Figure 4 presents the odds ratios for the 450K data with $K^{*}=\max(2,\hat{K})$; the heatmap in Figure S6.4 presents the odds ratios for the 27K data with $K^{*}=\max(2,\hat{K})$, and the odds ratios for$K=2$are given in Figures S7.5 and S7.6. Corresponding p-values are given in Tables S7.1, S7.2, and S7.3. Note that we excluded additional pairs from the 27K array analysis due to small DMP overlap with the 27K array.

Table S7.1 – P-values for 450K WGBS-Based Gene-Set Analyses, *K*=2 (Negative Base-10 Logarithmic Scale)

Table S7.2 – P-values for 450K WGBS-Based Gene-Set Analyses, *K*=*K** (Negative Base-10 Logarithmic Scale)

Table S7.3 – P-values for 27K WGBS-Based Gene-Set Analyses (Negative Base-10 Logarithmic Scale)

| Figure S7.1 – Clustering of Roadmap Epigenomics WGBS Specimens |  |
| --- | --- |
| Figure S7.2 – Deconvolution of Selected WGBS Tissues         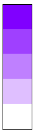 100%  0%  50% | Figure S7.3 – Network of WGBS Tissue Pairs Used for Gene Set Analysis |

Figure S7.4 – WGBS-Based Gene-Set Odds Ratios for 27K Data, *K*=*K*^*^


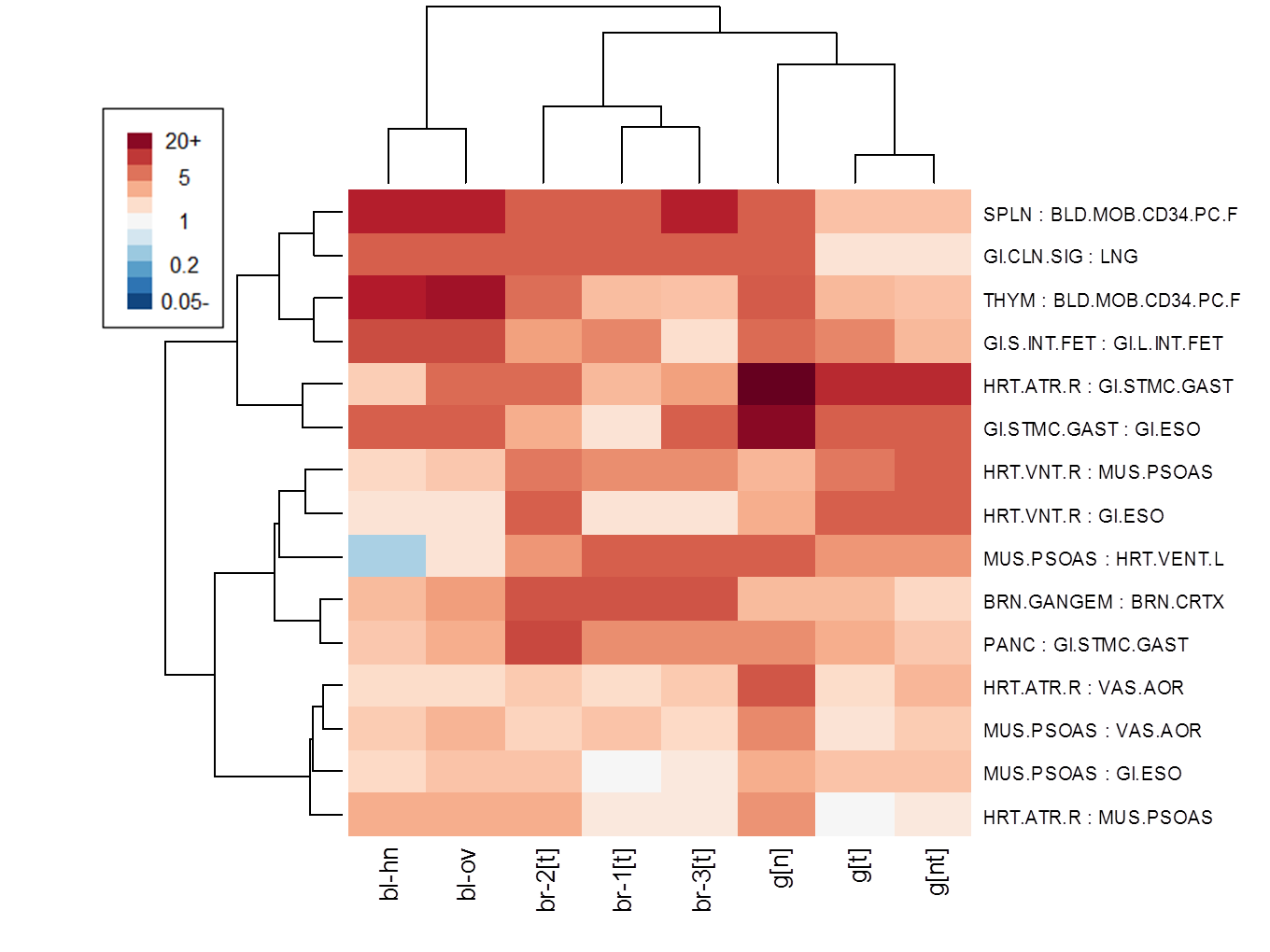


Figure S7.5 - WGBS-Based Gene-Set Odds Ratios for 450K Data, *K*=2


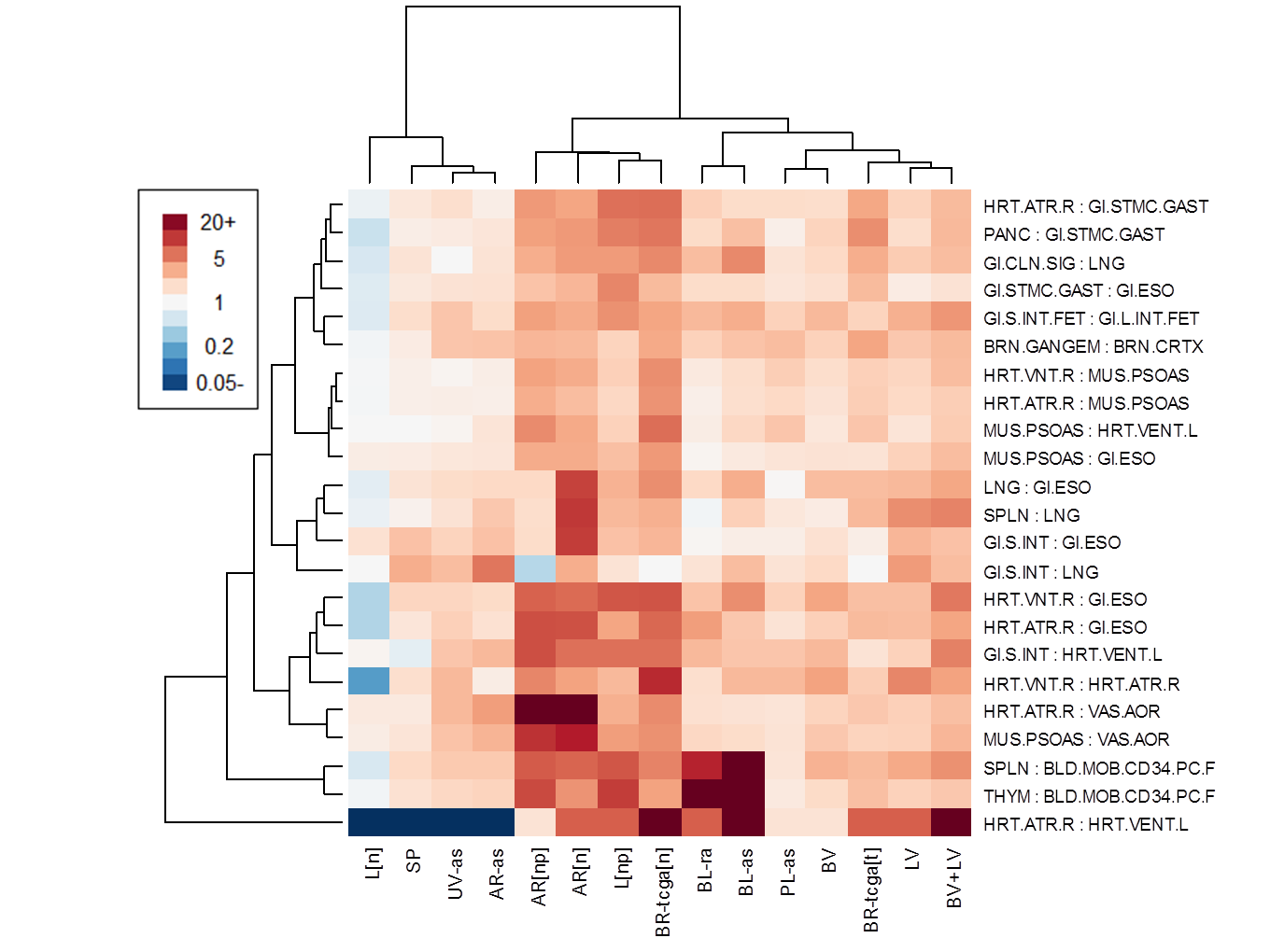


Figure S7.6 - WGBS-Based Gene-Set Odds Ratios for 27K Data, *K*=2


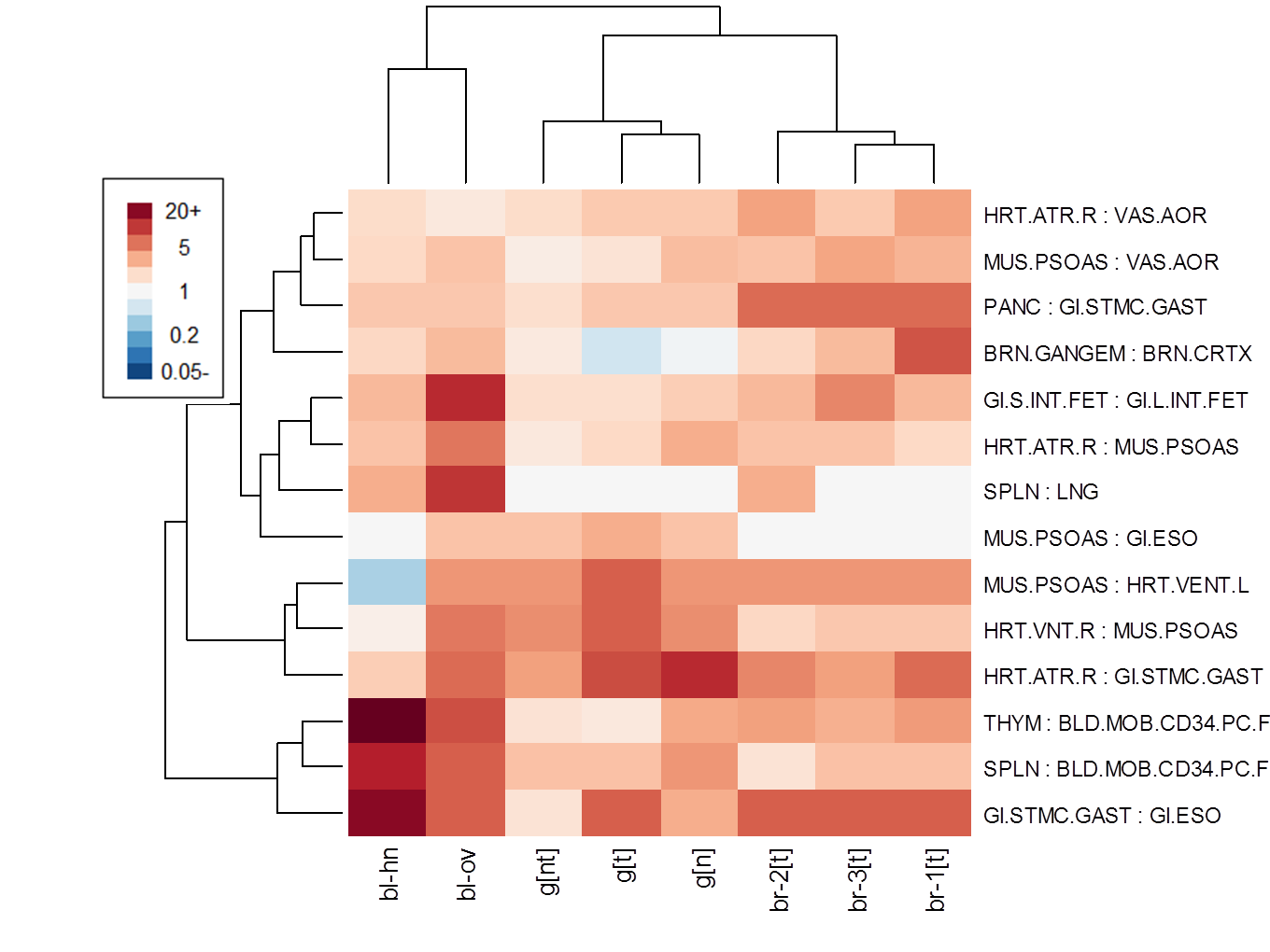


**Section S8 – Blood Specific Analysis**

Since reference data sets exist for blood, estimation of associations between phenotypic metadata and major types of leukocytes would typically employ the reference-based estimation of $\mathbf{\Omega}$ rather than the essentially unsupervised approach we have proposed in Section S2. However, in comparing the reference-based and reference-free approaches, two avenues of investigation emerge: (1) the extent to which the reference-based and reference-free approaches are consistent in their results; and (2) the extent to which the unsupervised approach may provide additional information on immune response and inflammation beyond associations with simply the major types of leukocytes, i.e. those existing in currently available reference sets. To this end, we have further analyzed the two 450K blood data sets, *BL-ra* and *BL-as*, estimating for each data set two sets of cell-type proportion matrices ($K=7$): $\mathbf{\Omega}_{0}$ (reference-based) and $\mathbf{\Omega}_{1}$ (reference-free). We used a common set of DMPs for each estimation procedure: using the ranked list of DMPs described in Section S6, we selected the top 5000 CpGs for differentiating major types of leukocytes in the Reinius data set, then complemented this set to $m=10,000$ with additional CpGs having highest variance across the samples within the dataset. We reasoned that this approach would provide enough information to estimate the major types of leukocytes, but might also provide additional information on more subtle immune and inflammation processes. With this set of CpGs, we fit $\mathbf{Y}=\mathbf{M}_{0}\mathbf{\Omega}_{0}^{\mathrm{T}}$ with essentially known $\mathbf{M}_{0}$ estimated from the Reinius data set[^4^](#_ENREF_4) while for the reference-free approach, we estimated $\mathbf{M}_{1}$ in the context of fitting $\mathbf{Y}=\mathbf{M}_{1}\mathbf{\Omega}_{1}^{\mathrm{T}}$, as described in Section S2.

We note that, in general, we do not anticipate $\mathbf{\Omega}_{0}$ and $\mathbf{\Omega}_{1}$ to be equal. The reason is that the unsupervised, reference-free approach will find only the major axes of variation within a given data set, not necessarily all relevant distinctions of major cell types. For example, if a data set consists of only two distinct immune profiles (with very little variation among the subjects sharing a profile), then the reference-free approach will typically find only two cell types, those corresponding to each profile. See Section S10 below for simulations demonstrating this phenomenon. However, $\mathbf{M}_{0}$ and $\mathbf{M}_{1}$ should be related to by a mixing matrix $\mathbf{\Psi}$ that reassigns the “correct” cell types to the unsupervised decomposition, i.e. $\mathbf{M}_{1}=\mathbf{M}_{0}\mathbf{\Psi}^{\mathrm{T}}$. The matrix $\mathbf{\Psi}$ can easily be obtained by constrained projection in the same manner that $\mathbf{\Omega}_{0}$ and $\mathbf{\Omega}_{1}$ are obtained, i.e. using essentially the procedure as step (2) in the algorithm of Section S2. Figures S8.1 and S8.2 depict the mixing matrices $\mathbf{\Psi}$ for *BL-ra* and *BL-as*, respectively, as clustering heatmaps. Table S8.1 provides the row sums of each $\mathbf{\Psi}$ matrix, indicating the presumed proportion of each column $\mathbf{M}_{1}$ of accounted for by $\mathbf{M}_{0}$; note that all values are close to one. Since$\mathbf{M}_{0}\mathbf{\Psi}^{\mathrm{T}}\mathbf{\Omega}_{1}^{\mathrm{T}}\approx\mathbf{M}_{1}\mathbf{\Omega}_{1}^{\mathrm{T}}\approx\mathbf{Y}\approx\mathbf{M}_{0}\mathbf{\Omega}_{0}^{\mathrm{T}}$, thus $\mathbf{\Omega}_{0}\approx\mathbf{\Omega}_{1}\mathbf{\Psi}$, it follows that phenotypic associations with $\mathbf{\Omega}_{0}$should match those with $\mathbf{\Omega}_{1}\mathbf{\Psi}$. Figures S8.3 and S8.4 demonstrate that the correspondence between $\mathbf{\Omega}_{0}$ and $\mathbf{\Omega}_{1}\mathbf{\Psi}$ is high for *BL-ra* and *BL-as*. Figures S8.5 and S8.6 compare regression coefficients for phenotypic associations with $\mathbf{\Omega}_{0}$ and $\mathbf{\Omega}_{1}\mathbf{\Psi}$; specifically, they compare the results of linear regression, where cell proportion (expressed as percentage points) was regressed on rheumatoid arthritis case status (*BL-ra*) or log_10_-arsenic (*BL-as*, adjusted for sex); 95% confidence intervals are shown for all coefficients. Phenotypic associations with the “re-mixed” $\mathbf{\Omega}_{1}\mathbf{\Psi}$ cell proportion estimates were remarkably similar to associations with the reference-based solution $\mathbf{\Omega}_{0}$, with only one notable reversal: in the *BL-ra* dataset, the relative magnitudes of CD4+ and CD8+ coefficients were reversed, but all were still significantly and negatively associated with rheumatoid arthritis status.

If additional information on immune function is available in our putative methylome matrix $\mathbf{M}_{1}$, then it should be evident in the residual matrix $\mathbf{M}_{1}-\mathbf{M}_{0}\mathbf{\Psi}^{\mathrm{T}}$, which reflects residual epigenetic information suggestive of cell-type heterogeneity but unaccounted for by the reference methylome $\mathbf{M}_{0}$. Figures S8.7 and S8.8 show plots of the residual row-variances $s_{j}^{(\Psi)2}$ of $\mathbf{M}_{1}-\mathbf{M}_{0}\mathbf{\Psi}^{\mathrm{T}}$ against the row-variances $s_{j}^{2}$ of $\mathbf{M}_{1}$. The plots reveal a cluster of CpGs with high $s_{j}^{2}$ (specifically,) and low to moderate $s_{j}^{(\Psi)2}$ (specifically, ), where and are the empirical distribution functions of $s_{j}^{2}$ and $s_{j}^{(\Psi)2}$ respectively. These are CpGs whose influence on variation within $\mathbf{\Omega}_{1}$ was accounted for by the reference methylome $\mathbf{M}_{0}$. The plots also reveal another cluster of CpGs whose row-variances are both high () and relatively similar, representing CpGs whose influence on variation within $\mathbf{\Omega}_{1}$ was *not* accounted for by $\mathbf{M}_{1}$. To assess the functional relevance of this latter set, we applied the methodology of Section S7 to dichotomous variables defined by these conditions, with results depicted in Figures S8.9 and S8.10. As anticipated, the residual methylomes $\mathbf{M}_{1}-\mathbf{M}_{0}\mathbf{\Psi}^{\mathrm{T}}$ displayed substantially diminished association with the DMPs based on Roadmap WGBS data, compared with the unadjusted methylomes $\mathbf{M}_{1}$.

To further understand the functional implications of our proposed method, we sought to compare our unsupervised method against reference-based deconvolution with respect to its ability to discern specific immune functions. We also compared both of these methods to *surrogate variable analysis* (SVA[^11^](#_ENREF_11)), a popular existing method used to adjust for cellular heterogeneity. For all methods, we set $K=7$ for to account for potential differences in estimating $K$. To interpret immune functional relevance, we used gene sets that identify processes involved in immune activation or regulation. Table S8.2 lists 14 sets of genes associated with immune activation or regulation, as identified by Qiagen’s T-Cell & B-Cell Activation PCR Array and compiled from seven sources[^12-18^](#_ENREF_12); we mapped CpG loci to the genes in each of these sets. Using the sets having at least 50 mapped CpGs, we tested the association of the resulting gene-set status with the dichotomous variables determined by $s_{j}^{2}>q_{0.75}(s^{2})$, where (as in Section S6) $s_{j}^{2}$ was the row variance of CpG $j$ in methylome $\mathbf{M}_{1}$, or its analog in the SVA or reference-based analysis, and $q_{0.75}(s^{2})$ was its corresponding upper quartile. Note that for the reference-based method we recomputed methylome $\mathbf{M}_{0}$ from $\mathbf{\Omega}_{0}$ and $\mathbf{Y}$, as is done in the removal-of-unwanted-variability (RUV) method. Figures S8.11 and S8.12 depict gene-set odds ratios for *BL-ra* and *BL-as*, respectively. Figure S8.11 shows that for *BL-ra*, both our proposed method and the reference-based method highlighted many functions related to T-cell differentiation, activation, and polarization, in particular processes affecting helper T-cells. In contrast, the SVA method highlighted fewer such functions (*T-cell differentiation* and *T-cell polarization*, with relatively weak significance and weak gene-set odds ratios). Only the reference-based method highlighted B-cell functions, which, though important, are less prominent than T-cells in the pathogenesis of rheumatoid arthritis. Figure S8.12 shows that for *BL-as*, only our proposed method highlighted any immune function. In particular, *Regulators of T-Cell Activators* and *T-Cell Polarization* were prominent, consistent with previously observed arsenic-related dysregulation of T-cells in the same Bangladeshi population[^19^](#_ENREF_19). In contrast with Figures S8.11 and S8.12, Figures S8.13 and S8.14 show gene-set odds-ratios for the distribution of limma-based p-values (as in Section S5 above), i.e. CpG-specific p-values for methylation associations with rheumatoid arthritis or arsenic exposure, adjusted for cell mixture; for each analysis we compared p-values in the lower-quartile with those of the other three quartiles. The results shown in these figures are generally consistent with results obtained from Kolmogorov-Smirnov tests (which require no p-value thresholds), although none of the Kolmogorov-Smirnov tests resulted in significant differences for our proposed method (in contrast to several for SVA and the reference-based method). For *BL-ra*, all ten gene sets showed differential limma-based significance in the SVA analysis, with four of them having odds ratios greater than 2.0; in contrast, only five of the gene-sets showed at most moderately differential limma-based significance using our approach, and only two gene-sets showed at most moderately differential limma-based significance using the reference-based approach. For *BL-as*, no gene sets showed strong differential limma-based significance using any of the methods. Finally, for *BL-ra* and *BL-as*, Figures S8.15 and S8.16 show the values of $\pi_{0}$ resulting from the limma analysis, reflecting the significance of associations with rheumatoid arthritis (*BL-ra*, Figure S8.15) or arsenic exposure (*BL-as*, Figure S8.16) after adjusting for either $\mathbf{\Omega}$, its equivalent matrix obtained using SVA, or the reference-based equivalent. All three methods produced similar values for *BL-as*; however, for *BL-ra*, our method produced a substantially larger value of $\pi_{0}$ than either SVA or the reference-based method. Taken together, these analyses show that SVA accounted for immune function principally in the “residual” associations evident from the CpG-specific limma analysis, with no functional interpretation evident from analysis of the methylomes implied by SVA. In contrast, many immune functions were evident from analysis of our proposed reference-free methylome matrix $\mathbf{M}$ or its reference-based equivalent; this was particularly evident for the *BL-as* dataset. Thus, compared with SVA, our method more accurately interpreted these immune functions as coordinated cellular processes rather than disparate, uncorrelated effects. We remark that although both SVA and the reference-based method resulted in similarly low values of $\pi_{0}$(Figure S8.15), the limma analysis highlighted many more immune functions using SVA than those highlighted using either our proposed method or the reference-based method. Note that when we increased $K$ to 33, the value properly estimated by SVA, $\pi_{0}$rose to 0.55 and the corresponding gene-set results that were more similar to those obtained from our method; this suggests that SVA produces results similar to those of our method, though using many more degrees-of-freedom to represent the cellular heterogeneity. It is interesting to note that our analysis highlighted several immune processes that are supported by the arsenic exposure literature, but not evident from the SVA or reference-based analysis. In summary, our method may produce results similar to those obtained from SVA, but using potentially fewer degrees of freedom, and reference-free deconvolution may highlight immune functionality not evident using reference-based deconvolution alone.

Table S8.1 – Row Sums of $\mathbf{\Psi}$

Table S8.2 – Immune Activation/Regulation Gene Sets

Source: Qiagen Corp., <http://www.sabiosciences.com/rt_pcr_product/HTML/PAHS-053Z.html>

| Figure S8.1 – Mixing Matrix $\mathbf{\Psi}$ for *BL-ra* Dataset       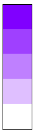 100%  0%  50% | Figure S8.2 – Mixing Matrix $\mathbf{\Psi}$ for *BL-as* Dataset       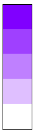 100%  0%  50% |
| --- | --- |
| Figure S8.3 – Comparison of $\mathbf{\Omega}_{0}$ and $\mathbf{\Omega}_{1}\mathbf{\Psi}$ for *BL-ra* Dataset | Figure S8.4 – Comparison of $\mathbf{\Omega}_{0}$ and $\mathbf{\Omega}_{1}\mathbf{\Psi}$ for *BL-as* Dataset |
| Figure S8.5 – Comparison of $\mathbf{\Omega}_{0}$ and $\mathbf{\Omega}_{1}\mathbf{\Psi}$ for Case/Control Association in *BL-ra* Dataset    Note: y-axis shows regression coefficients with 95% confidence intervals. | Figure S8.6 – Comparison of $\mathbf{\Omega}_{0}$ and $\mathbf{\Omega}_{1}\mathbf{\Psi}$ for Log-Arsenic Association in *BL-ra* Dataset    Note: y-axis shows regression coefficients with 95% confidence intervals. |

| Figure S8.7 – Unadjusted Reference-Free Row-Variance $s_{j}^{2}$ vs. Reference-Adjusted Row-Variance $s_{j}^{(\Psi)2}$: *BR-ra* Dataset  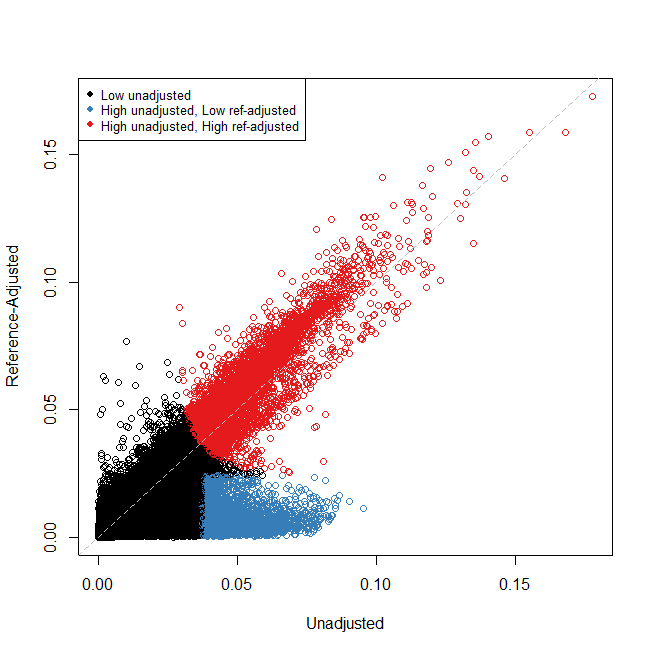 | Figure S8.8 – Unadjusted Reference-Free Row-Variance $s_{j}^{2}$ vs. Reference-Adjusted Row-Variance $s_{j}^{(\Psi)2}$: *BR-as* Dataset  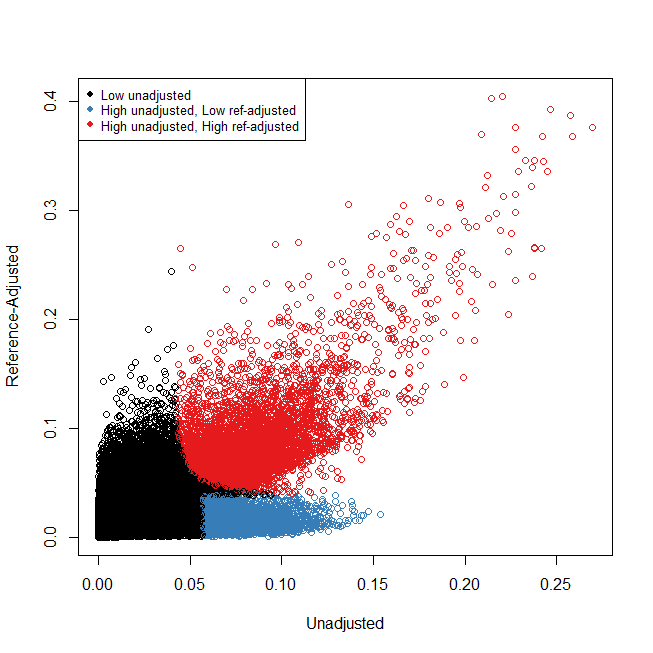 |
| --- | --- |
| Figure S8.9 – WGBS-Based Gene-Set Odds Ratios: High Unadjusted, Low Reference-Adjusted | Figure S8.10 – WGBS-Based Gene-Set Odds Ratios: High Unadjusted, High Reference-Adjusted |
| Figure S8.11 – Gene Set Odds Ratios Comparing Methylome Variability Across Gene Sets: *BR-ra*    Odds ratios represent enrichment of indicated gene set for Methylome rows having $s_{j}^{2}>q_{0.75}(s^{2})$. Gene set enrichment significance assessed by Fisher’s Test: * p<0.05, ** p<0.01, *** p<0.001. | Figure S8.12 – Gene Set Odds Ratios Comparing Methylome Variability Across Gene Sets: *BR-as*    Odds ratios represent enrichment of indicated gene set for Methylome rows having $s_{j}^{2}>q_{0.75}(s^{2})$. Gene set enrichment significance assessed by Fisher’s Test: * p<0.05, ** p<0.01, *** p<0.001. |
| Figure S8.13 – Gene Set Odds Ratios Comparing Mixture-Adjusted Nominal CpG-Specific P-Values Across Gene Sets: *BR-ra*    Odds ratios represent enrichment of indicated gene set for CpGs whose rheumatoid arthritis p-values, adjusted for cell type heterogeneity, were in the lower quartile. Gene set enrichment significance assessed by Fisher’s Test: * p<0.05, ** p<0.01, *** p<0.001. | Figure S8.14 – Gene Set Odds Ratios Comparing Mixture-Adjusted Nominal CpG-Specific P-Values Across Gene Sets: *BR-as*    Odds ratios represent enrichment of indicated gene set for CpGs whose arsenic exposure p-values, adjusted for cell type heterogeneity, were in the lower quartile. Gene set enrichment significance assessed by Fisher’s Test: * p<0.05, ** p<0.01, *** p<0.001. |

| Figure S8.15 – Estimated Proportion of Null Arthritis Associations in *BL-ra* After Adjusting for Cell Type Heterogeneity | Figure S8.16 – Estimated Proportion of Null Arsenic Exposure Associations in *BL-as* After Adjusting for Cell Type Heterogeneity |
| --- | --- |

**Section S9 – Analysis of Normal vs. Pathological Tissue**

Figure S7.2 above displays the cell-proportion matrix $\mathbf{\Omega}$ from the decomposition $\mathbf{Y}=\mathbf{M\Omega}^{\mathrm{T}}$ of Roadmap WGBS data; in the figure, the $K=6$ putative cell types are labeled according to reasonable anatomical interpretations of the resultant groupings. We projected Infinium data from each of the three datasets sets *g[nt]*, *AR[np]*, and *L[np]* onto the profile matrix $\mathbf{M}$ obtained from the WGBS data (as in step (1) of Section S2), thus obtaining specimen-specific proportions $\mathbf{\Omega}$ for each of the cell types determined from the WGBS data. Figures S9.1 through S9.3 compare the average resulting cell proportion for normal tissue with the corresponding average for pathological tissues. Note that separate averages were computed for atherosclerotic aorta and atherosclerotic carotid (*AR[np]*), and for alcohol-related cirrhotic liver and cirrhotic liver related to viral infection (*L[np]*).

| Figure S9.1 – Comparison of Roadmap WGBS cell types: Normal vs. Tumor in *g[nt]*    Average cell proportion for types based on Roadmap Epigenomics WGBS data (see Figure S7.2 for depiction of types indicated as text in this figure). | Figure S9.2 – Comparison of Roadmap WGBS cell types: Normal vs. Tumor in *AR[nt]*    Average cell proportion for types based on Roadmap Epigenomics WGBS data (see Figure S7.2 for depiction of types indicated as text in this figure). |
| --- | --- |
| Figure S9.3– Comparison of Roadmap WGBS cell types: Normal vs. Tumor in *L[nt]*    Average cell proportion for types based on Roadmap Epigenomics WGBS data (see Figure S7.2 for depiction of types indicated as text in this figure). |  |

**Section S10 – Simulations**

We conducted simulations to compare the behavior of our proposed deconvolution method with other methods. We first remark that the principal desirable property of any unsupervised deconvolution method $\mathbf{Y}=\mathbf{M\Omega}^{\mathrm{T}}$ is low residual error $RMSE=\sum_{j=1}^{m} \left\|\mathbf{y}_{j}^{(r)}-\mathbf{\Omega\mu}_{j}\right\|^{2}$ as well as high R^2^ values for each of the individual true cell types[^20^](#_ENREF_20). However, in a context where the factors $\mathbf{M}$ and $\mathbf{\Omega}$ are assumed to have a biological meaning, deconvolution estimates of both $\mathbf{M}$ and $\mathbf{\Omega}$ should lie as close to their true data-generating values as possible. In particular, the estimated number $\hat{K}$ of cell types should approximate the true number $K$. However, even when $K$ can be accurately estimated, solutions $\mathbf{M}$ and $\mathbf{\Omega}$ will be unique only up to rotation, a limitation that is well known in the factor analysis literature[^21^](#_ENREF_21); thus constraints are often imposed so that the solution reflects prior assumptions about the data generation process. This was one motivation for using independent components analysis as the basis of ICSA[^22^](#_ENREF_22). Our proposed method imposes constraints $0\leq\mu_{jk}\leq1$, $0\leq\omega_{ik}\leq1$, and $\sum_{k=1}^{K} \omega_{ik}\leq1$. A popular and accessible alternative, non-negative matrix factorization (NNMF) imposes weaker constraints $0\leq\mu_{jk}$ and $0\leq\omega_{ik}$ only. Other simple alternatives include factor analysis (FA) and its related method, principal components analysis (PCA), both which effectively impose no constraint on the solution other than orthogonality of the mixture coefficients (an unnatural assumption). In this simulation we compare our proposed method “convex factorization” with NNMF and PCA, where the latter was conducted both on the standardized z-scores and the unstandardized data (for justification of the latter, see Houseman et al., 2014). Note that the PCA solutions were obtained by singular value decomposition, taking the first $K$ singular right vectors (the totality of which form an $n\times n$ orthogonal matrix).

We also remark that in an unsupervised setting, the solutions present no obvious labeling of the $K$ factors. Thus, in a simulation study where solutions are compared to data-generating values having presumed biological interpretation, any solution must be aligned to its corresponding data generating value. Similar to Section S8 above, an obvious way to align biological truth $\mathbf{\Omega}_{0}$ with its corresponding estimate $\mathbf{\Omega}_{1}$ (assumed to have the correct number $K$ of columns) is to seek a matrix $\mathbf{A}$ that minimizes$||\mathbf{\Omega}_{1}-\mathbf{\Omega}_{0}\mathbf{A}||$, i.e $\mathbf{A}=(\mathbf{\Omega}_{0}^{\mathrm{T}}\mathbf{\Omega}_{0})^{=1}\mathbf{\Omega}_{0}^{\mathrm{T}}\mathbf{\Omega}_{1}$. The ideal such matrix is a (potentially improper) rotation matrix, i.e. a matrix $\mathbf{A}$ such that $\det(\mathbf{A})=\pm1$ (note that since $\mathbf{A}$ is not symmetric, its determinant need not be positive). Thus methods that tend to produce matrices $\mathbf{A}$ with log-absolute-determinants close to zero are preferred, i.e. those that produce small values of the absolute-log-absolute-determinant,$\Delta=|\log|\det(\mathbf{A})||$.

If a solution obeys biological constraints, at least approximately, then ideally $\mathbf{A}$ should actually be a *Markov* matrix, i.e. a matrix $\mathbf{P}$ whose values lie in the unit interval and whose rows sum to one. Ideally $\mathbf{P}$ should not spread one true cell type across many estimated cell types, although it may collapse several true types to one putative type if there is insufficient information to distinguish the types (as we demonstrate below). This can be assessed by examining the entropy of the rows $l$ of $\mathbf{P}$, $H_{l}(\mathbf{P})=-\sum_{k} p_{lk}\log(p_{lk})$, where the row-specific sum (corresponding to a particular true cell type) is taken over all of its (column) entries and $0\log(0)$ is assumed to be its asymptotic limit, zero. When $\mathbf{P}$ is the identity matix (the ideal), $H_{l}(\mathbf{P})=0$ for all columns. At the other extreme, when the solution spreads a true cell type uniformly across all putative cell types, then . We define *total entropy* as $\sum_{l=1}^{K} H_{l}(\mathbf{P})$ and *weighted entropy* as $\sum_{l=1}^{K} (n^{-1}\sum_{i=1}^{n} \omega_{il})H_{l}(\mathbf{P})$, where the latter quantity weights each cell-type-specific entropy by its mean value in the population, i.e. emphasizes cell types that are more prevalent in the population.

*Data Generation*

We sought to make our simulation study as realistic as possible, within computational constraints. For the “true” proportions, we obtained reference-based cell proportions from the rheumatoid arthritis data set *BL-ra*, then used maximum likelihood to fit them to the following Dirichlet distribution model:

$f(\mathbf{\omega}_{i})=\frac{\prod_{k=1}^{8} \omega_{ik}^{\alpha_{k}(x_{i})}\Gamma(\sum_{k=1}^{8} \alpha_{k}(x_{i}))}{\prod_{k=1}^{8} \Gamma(\alpha_{k}(x_{i}))}, \alpha_{k}(x_{i})=\exp(\varsigma_{k0}+\varsigma_{k1}x_{i})$, (S10.1)

where we considered the seven types of leukocytes profiled in the Reinius reference data set along with a remainder term (so that the proportions sum to one), and $\mathbf{\omega}_{i}=(\omega_{ik})$was the cell proportion vector for subject $i$ with arthritis status $x_{i}$ (0 for control, 1 for case). All simulations were based on the fitted coefficients. For each simulation scenario, we generated cell proportions for $n=100$ subjects either by uniformly setting $x_{i}=0$ (corresponding to healthy subjects only), or else generating equal numbers of cases and controls (as described in more detail below). The corresponding methylation matrix $\mathbf{Y}$ was generated by first calculating the “true” methylation values $\mathbf{Y}_{0}=\mathbf{M\Omega}_{0}^{\mathrm{T}}$ , $\mathbf{Y}_{0}=(y_{0ji})$, using $\mathbf{M}_{0}$ obtained from the Reinius reference data set (where we used either the 500 or 5000 most discriminating CpGs), then generated “noisy” methylation values $\mathbf{Y}=(y_{ji})$ (i.e. incorporating microarray measurement error) using a beta distribution with parameters $y_{0ji}\tau$ and $(1-y_{0ji})\tau$ [leading to mean $y_{0ji}$ and variance$y_{0ji}(1-y_{0ji})(\tau+1)^{-1}$]. As described below, we set $\tau=100$ or $\tau=200$, which for methylation values = 0.5, corresponded to standard deviations of 0.050 and 0.035, respectively (i.e. about the level of error on the 450K array). For each scenario, we simulated 500 data sets.

Table S10.1 below describes the specific scenarios. Most of them involved straightforward adaptations of the Dirichlet parameters described above, in order to assess the impact of different scales of variation or covariation. However, Scenarios 8 and 9 were slightly more complex. In order to assess the effect of cellular subtypes on estimation of $K$, we generated profiles for several distinct subtypes of neutrophils and CD4+ T cells. For Scenario 8, we added 4 subtypes of neutrophils and 6 subtypes of CD4+ T-cells, each of these types characterized by 50 additional methylated loci (unmethylated for all other types and subtypes). Thus 500 artificial loci were added. Cell proportions were generated by splitting the neutrophil and CD4+ T cell proportions, each via a separate Dirichlet variable. For neutrophils, the Dirichlet parameters were 8 for the “naive” cell type and 1 for the other subtypes (corresponding to 0.667 probability of a neutrophil being in its naïve state), and for CD4+ T cells, the Dirichlet parameters were 9 for the “naïve” cell type and 1 for the other types (corresponding to a 0.60 probability of a CD4+ T cell remaining in its “naïve” state). Scenario 9 was similar to Scenario 8, with 10 added neutrophil subtypes and 20 added CD4+ T cell subtypes, each subtype corresponding to 25 methylated loci (adding 750 artificial loci to the simulation).

For scenario 1, Figure S10.1 shows the distribution of cell-type specific standard deviations of $\mathbf{\omega}_{i}=(\omega_{ik})$ computed separately on each of 500 simulated data sets, while Figure S10.2 shows a scatter-plot of the two most common cell types, neutrophils and CD4+ T-cells. Note the fact that neutrophils and CD4+ T-cells proportions are dependent, i.e. they are non-orthogonal.

Table S10.1 – Summary of Simulation Scenarios

| Scenario | # Loci | Microarray Prec. $\tau$ | Model Description |
| --- | --- | --- | --- |
| 1 | 5000 | 100 | Cell proportions based only on *BL-ra* controls: $n=100$ subjects simulated with $\alpha_{k}=\exp(\hat{\varsigma}_{k0})$from equation (S10.1) above. |
| 2 | 5000 | 200 |
| 3 | 500 | 100 |
| 4 | 500 | 200 |
| 5 | 500 | 200 | Cell proportions based on *BL-ra* cases and controls but with greater precision: $n=50$ with $\alpha_{k}=10^{c}\exp(\hat{\varsigma}_{k0})$, $n=50$ with ; $c=1$(Sc. 5) or $c=2$(Sc. 6). |
| 6 | 500 | 200 |
| 7 | 500 | 200 | As in Sc. 4 but with $\alpha_{k}=10\exp(\hat{\varsigma}_{k0})$[greater precision]. |
| 8 | 500+500 | 200 | As in Sc. 4, but with Neutrophil and CD4+ cell proportions distributed over subtypes; Sc. 9 had more subtypes than Sc. 8. |
| 9 | 500+750 | 200 |

| Figure S10.2 – Cell-type standard deviations for Scenarios 1 and 2 | Figure S10.2 – Simulated CD4+T vs. Neutrophil proportions for Scenarios 1 and 2  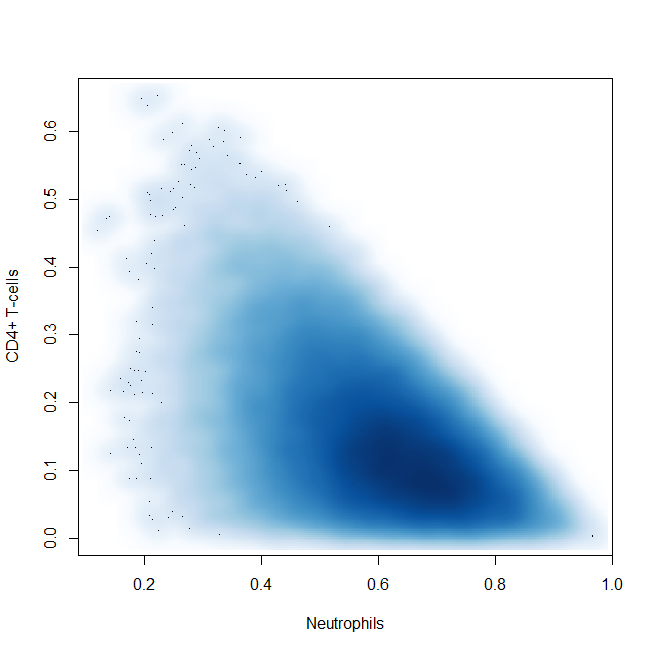 |
| --- | --- |

*Evaluation of Methods*

As motivated above, for Scenarios 1 and 2, we calculated $RMSE=\sum_{j=1}^{m} \left\|\mathbf{y}_{j}^{(r)}-\mathbf{\Omega\mu}_{j}\right\|^{2}$ as well as R^2^ values for each of the individual true cell types. In addition, we calculated $\Delta=|\log|\det(\mathbf{A})||$ for each method, where for each simulated data set, $\mathbf{A}$ was obtained by linear projection (i.e. linear regression). Note that in calculating $\mathbf{A}$ for the PCA methods, we permitted re-centering by intercept terms (i.e. estimated an affine transformation), since the principal component scores tended to center around zero and thus did not obey the biological constraints imposed upon $\mathbf{\omega}_{i}$.; for the convex and NNMF methods, we constrained the intercepts to be zero. To measure the relative singularity of $\mathbf{A}$, (another measure of the extent to $\mathbf{A}$ which deviated from a rotation matrix) we also calculated its condition number (maximum singular value divided by minimum singular value). Finally, for each data set and each method, we obtained a Markov matrix $\mathbf{P}$ by minimizing $||\mathbf{\Omega}_{1}-\mathbf{\Omega}_{0}\mathbf{P}||$, with appropriate Markov constraints, and subsequently computed total and weighted entropy (as defined above).

We also sought to compare different methods of estimating$K$(Scenarios 3-9). In addition to the proposed bootstrap-based method, we applied a method that applies random matrix theory (RMT) to the residual matrix to estimate the rank of its probabilistic support. The basic method was originally proposed for ISVA[^22^](#_ENREF_22), and later adapted to the present problem[^10^](#_ENREF_10), [^23^](#_ENREF_23). We also applied an ad-hoc method that represents a common approach in factor analysis, where we fit an unstandardized factor analysis model to $\mathbf{Y}$ and set $K$ equal to the minimum value that produced an insignificant goodness-of-fit p-value (p>0.05).

*Results*

Figures S10.3 and S10.4 show the RMSE for scenarios 1 and 2, which were based on leukocyte proportions from the *BL-ra* data set assuming different levels of microarray precision (less precise $\tau=100$in Scenario 1 vs. more precise $\tau=200$in Scenario 2). These figures show that PCA typically produced a better fit to the underlying observed data $\mathbf{Y}$. This was expected, since PCA entails no constraints on the solution. R^2^ values showed a very similar pattern (figures not shown), reinforcing the superiority of PCA-based methods in statistical terms. However, among the constrained solutions, the convex method tended to provide a better fit. Figure S10.5 and S10.6 show the absolute values of the log-absolute-determinant statistics $\Delta$ for Scenarios 1 and 2, while Figures S10.7 and S10.8 show the corresponding condition numbers. Typically the values of $\Delta$ were smaller for our proposed method, although the unstandardized PCA often produced values of $\Delta$ almost as small (paired Wilcoxon p<10^-5^ for all comparisons of *convex* vs. other methods, except for Scenario 1, *PCA(2)* vs. *convex*, where the median value of $\Delta$ was slightly smaller for *PCA(2)*, and p=0.027). As measured by condition number, standardized PCA tended to provide the least singular transformations between true coefficients and estimates (paired Wilcoxon p<10^-7^ for all comparisons of *convex* vs. other methods), but our proposed method typically produced condition numbers almost as small. Finally, as shown in Figures S10.9-12, our proposed convex method produced unequivocally smaller entropy statistics than the other methods (paired Wilcoxon p<10^-16^ for all comparisons of *convex* with other methods). Taken together, Scenarios 1 and 2 show that our proposed method optimizes the tradeoff between fit and interpretability: while the unconstrained methods produce slightly better statistical fit to the data, the optimal fit comes at the expense of coefficients that are difficult to assign biological significance.

| Figure S10.3 – RMSE for Scenario 1 | Figure S10.3 – RMSE for Scenario 2 |
| --- | --- |

| Figure S10.5 – Log-absolute-determinants for Scenario 1 | Figure S10.6 – Log-absolute-determinants for Scenario 2 |
| --- | --- |

| Figure S10.7 – Log-condition-numbers for Scenario 1 | Figure S10.8 – Log-condition-numbers for Scenario 2 |
| --- | --- |

| Figure S10.9 – Total Entropy for Scenario 1 | Figure S10.10 – Total Entropy for Scenario 2 |
| --- | --- |

| Figure S10.11 – Weighted Entropy for Scenario 1 | Figure S10.12 – Weighted Entropy for Scenario 2 |
| --- | --- |

Scenarios 3 and 4 were designed to provide an initial comparison of different methods of estimating$K$. These scenarios differ from Scenarios 1 and 2 only in the number of CpGs used, with the lower number selected to decrease computation time. Results are shown in Figures S10.13 and S1014. Compared with RMT, our proposed bootstrap method tended to estimate $K$closer to its true value of 7, although both methods always underestimated the truth. The ad-hoc factor analysis method tended to overestimate $K$, and additionally lacked specificity.

| Figure S10.13 – Estimation of *K* (Scenario 3)  Mean $\hat{K}$: 7.7 (FA-sig), 3.9 (RMT), 4.6 (convex-boot). | Figure S10.14 – Estimation of *K* (Scenario 4)    Mean $\hat{K}$: 6.6 (FA-sig), 4.6 (RMT), 5.2 (convex-boot). |
| --- | --- |

Related to the problem of under-estimating $K$, our reviewers asked us to explain why our method does not reliably reproduce *all* constituent cell types. To address their quite reasonable request, we added Scenarios 5-7, which represent hypothetical extreme situations and demonstrate the limiting behavior of reference-free deconvolution in general. As shown in Figures S10.15 and S10.16, Scenarios 5 and 6 involved two very precise clusters of subjects. As shown in the corresponding Figures S10.17 and S10.18, our convex-bootstrap method tended to find only 2 or 3 classes, with $K=2$ occurring more frequently in Scenario 6, which had tighter clusters. The factor analysis solution again lacked specificity, while the RMT method tended to find values of $K$ less than or equal to the value found by our proposed method. We have argued previously that, in the context of reference-free estimation of cell proportions, RMT may estimate the number of axes of variation rather than the number of cell types[^10^](#_ENREF_10), and these simulations illustrate the point. In particular, unsupervised methods can find only the axes of variation supported by the data set at hand, not the full range of biological variability across all human populations. This is further demonstrated by Scenario 7, similar to Scenario 4, but with smaller population variance (Figure S10.19). Our method almost always estimated $K=3$, while RMT estimated $K=2$ or 3, and the factor analysis method again lacked specificity (Figure S10.20).

As a final remark, we note that when $K$ is estimated to be much lower than the true number of classes, due to lack of variability within the data set, we would not expect mixture coefficients that represent a simple rotation of the truth. Rather, we would expect the true cell types to collapse somewhat. However, we would still hope for relatively specific classes; in scenarios 5 through 7, total and weighted entropy comparisons between our proposed method and NNMF resulted in figures similar to Figure S10.9-12 above. Figure S10.21 illustrates the differences in total entropy from our proposed method across Scenarios 4, 5, and 6; thus the figure represents a progression from indistinct groups to very tight clusters. As evident from the figure, entropy increases with tighter clusters because the data distinguish the subpopulations (cases vs. controls) rather than biologically interpretable cell types.

| Figure S10.15 – Simulated CD4+T vs. Neutrophil proportions for Scenario 5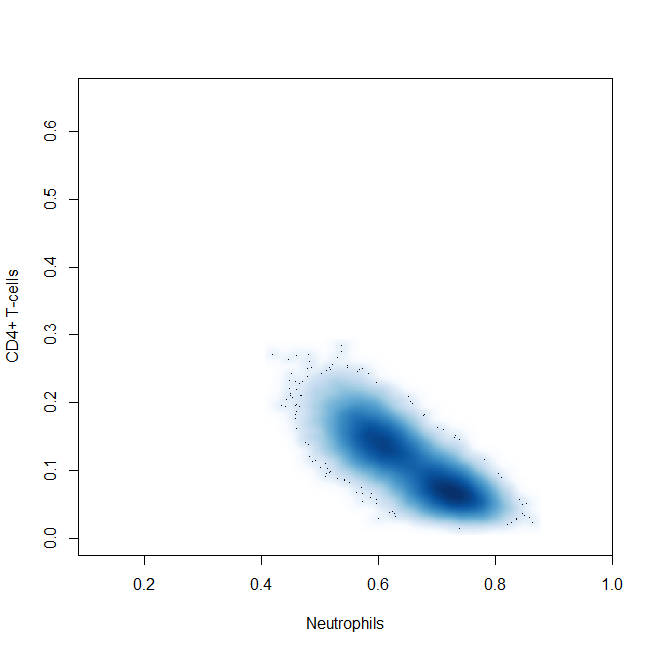  Note that the figure scale is identical with that of Figure S10.2. | Figure S10.16 – Simulated CD4+T vs. Neutrophil proportions for Scenario 6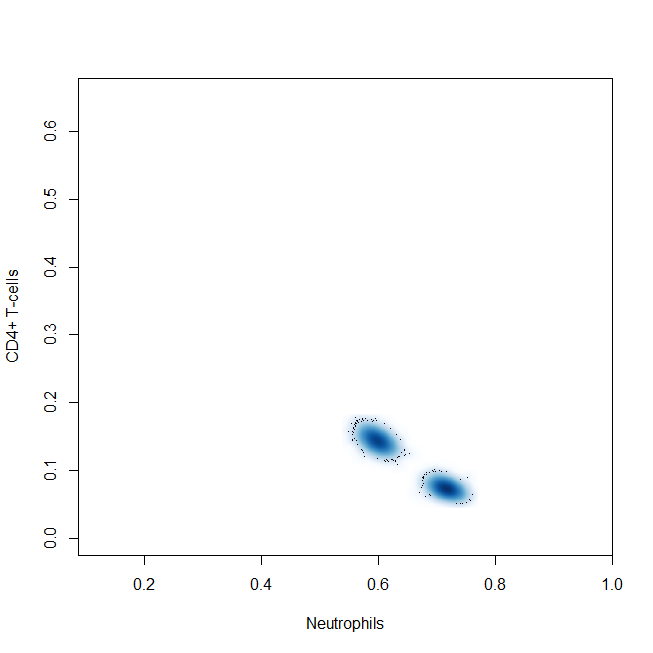  Note that the figure scale is identical with that of Figure S10.2. |
| --- | --- |
| Figure S10.17 – Estimation of *K* (Scenario 5)    Mean $\hat{K}$: 6.8 (FA-sig), 2.3 (RMT), 3.0 (convex-boot). | Figure S10.18 – Estimation of *K* (Scenario 6)    Mean $\hat{K}$: 4.8 (FA-sig), 1.0 (RMT), 2.0 (convex-boot). |

| Figure S10.19 – Simulated CD4+T vs. Neutrophil proportions for Scenario 7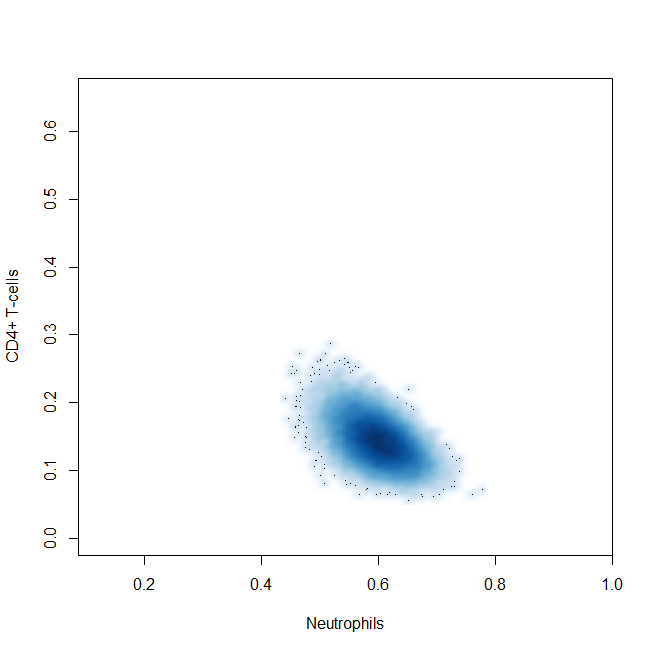  Note that the figure scale is identical with that of Figure S10.2. | Figure S10.20 – Estimation of *K* (Scenario 7)  Mean $\hat{K}$: 6.5 (FA-sig), 2.6 (RMT), 3.0 (convex-boot). |
| --- | --- |

| Figure S10.21 – Comparison of total entropy from convex algorithm across Scenarios 4, 5, and 6 |  |
| --- | --- |

Finally, Scenarios 8 and 9 demonstrate the impact of cellular subtypes on estimation of $K$. As shown in Figures 10, all methods produced estimates that tended to be larger than those of previous simulations. These results illustrate that heterogeneity in cellular subtypes can result in larger estimates of $K$.

| Figure S10.22 – Estimation of *K* (Scenario 8)    Mean $\hat{K}$: 6.2 (FA-sig), 7.9 (RMT), 8.8 (convex-boot). | Figure S10.23 – Estimation of *K* (Scenario 9)  Mean $\hat{K}$: 9.5 (FA-sig), 10.1 (RMT), 7.2 (convex-boot). |
| --- | --- |

As a final remark, we note that a bootstrap procedure very similar to the one we have proposed for our convex deconvolution could be applied in the setting of ordinary NNMF. However, we have observed that the NNMF procedure typically took over four times as long to run; thus our approach may be more computationally efficient.

**References**

1. Goldfarb, D. & Idnani, A. A numerically stable dual method for solving strictly convex quadratic programs. *Mathematical programming* **27**, 1-33 (1983).

2. Houseman, E.A. et al. Model-based clustering of DNA methylation array data: a recursive-partitioning algorithm for high-dimensional data arising as a mixture of beta distributions. *BMC bioinformatics* **9**, 365 (2008).

3. Smyth, G.K. Linear models and empirical bayes methods for assessing differential expression in microarray experiments. *Stat Appl Genet Mol Biol* **3**, 3 (2004).

4. Reinius, L.E. et al. Differential DNA methylation in purified human blood cells: implications for cell lineage and studies on disease susceptibility. *PloS one* **7**, e41361 (2012).

5. Teschendorff, A.E. et al. A beta-mixture quantile normalization method for correcting probe design bias in Illumina Infinium 450 k DNA methylation data. *Bioinformatics* **29**, 189-196 (2013).

6. Bracken, A.P., Dietrich, N., Pasini, D., Hansen, K.H. & Helin, K. Genome-wide mapping of Polycomb target genes unravels their roles in cell fate transitions. *Genes & development* **20**, 1123-1136 (2006).

7. Lee, T.I. et al. Control of developmental regulators by Polycomb in human embryonic stem cells. *Cell* **125**, 301-313 (2006).

8. Schlesinger, Y. et al. Polycomb-mediated methylation on Lys27 of histone H3 pre-marks genes for de novo methylation in cancer. *Nature genetics* **39**, 232-236 (2006).

9. Squazzo, S.L. et al. Suz12 binds to silenced regions of the genome in a cell-type-specific manner. *Genome research* **16**, 890-900 (2006).

10. Houseman, E.A., Kelsey, K.T., Wiencke, J.K. & Marsit, C.J. Cell-composition effects in the analysis of DNA methylation array data: a mathematical perspective. *BMC bioinformatics* **16** (2015).

11. Leek, J.T. & Storey, J.D. Capturing heterogeneity in gene expression studies by surrogate variable analysis. *PLoS Genet* **3**, 1724-1735 (2007).

12. Criscione, L.G. & Pisetsky, D.S. B lymphocytes and systemic lupus erythematosus. *Current rheumatology reports* **5**, 264-269 (2003).

13. Tseng, S.Y. & Dustin, M.L. T-cell activation: a multidimensional signaling network. *Current opinion in cell biology* **14**, 575-580 (2002).

14. Jaeckel, E. Animal models of autoimmune hepatitis. *Seminars in liver disease* **22**, 325-338 (2002).

15. Poindexter, N.J., Sahin, A., Hunt, K.K. & Grimm, E.A. Analysis of dendritic cells in tumor-free and tumor-containing sentinel lymph nodes from patients with breast cancer. *Breast cancer research : BCR* **6**, R408-415 (2004).

16. Ragde, H., Cavanagh, W.A. & Tjoa, B.A. Dendritic cell based vaccines: progress in immunotherapy studies for prostate cancer. *The Journal of urology* **172**, 2532-2538 (2004).

17. Tedder, T.F., Poe, J.C., Fujimoto, M., Haas, K.M. & Sato, S. The CD19-CD21 signal transduction complex of B lymphocytes regulates the balance between health and autoimmune disease: systemic sclerosis as a model system. *Current directions in autoimmunity* **8**, 55-90 (2005).

18. Wang, E., Panelli, M.C. & Marincola, F.M. Understanding the response to immunotherapy in humans. *Springer seminars in immunopathology* **27**, 105-117 (2005).

19. Ahmed, S. et al. Arsenic-associated oxidative stress, inflammation, and immune disruption in human placenta and cord blood. *Environ Health Perspect* **119**, 258-264 (2011).

20. Rahmani, E. et al. Sparse PCA corrects for cell type heterogeneity in epigenome-wide association studies. *Nature methods* **13**, 443-445 (2016).

21. Russell, D.W. In search of underlying dimensions: The use (and abuse) of factor analysis in Personality and Social Psychology Bulletin. *Pers Soc Psychol B* **28**, 1629-1646 (2002).

22. Teschendorff, A.E., Zhuang, J. & Widschwendter, M. Independent surrogate variable analysis to deconvolve confounding factors in large-scale microarray profiling studies. *Bioinformatics* **27**, 1496-1505 (2011).

23. Houseman, E.A. & Ince, T.A. Normal cell-type epigenetics and breast cancer classification: a case study of cell mixture-adjusted analysis of DNA methylation data from tumors. *Cancer informatics* **13**, 53-64 (2014).
